# Evaluation scale and behavioral model construction for intention to use postpartum exercise rehabilitation mobile application based on user experience

**DOI:** 10.3389/fpsyg.2025.1575049

**Published:** 2025-05-30

**Authors:** Zhiyuan Wang, Zheng Wang, Rong Deng, Meng Xia, Liming Huang

**Affiliations:** ^1^School of Design, Jiangnan University, Wuxi, China; ^2^School of Rehabilitation Medicine, Nanjing Medical University, Nanjing, China

**Keywords:** exercise rehabilitation, postpartum, maternal wellbeing, digital health, mobile health, intention to use, linear regression

## Abstract

**Background:**

Physical exercise is a widely recognized and practical approach to postnatal rehabilitation. In recent years, its delivery through mobile applications has become increasingly prevalent due to their accessibility and affordability. These mobile applications are crucial in supporting postpartum women in restoring physical and mental well-being and promoting sustainable health behaviors. The intention to use such mobile applications is a key determinant of user behavior and offers valuable guidance for designing and improving digital health services. Despite this, current research has largely overlooked the intention to use postpartum exercise rehabilitation mobile applications from the user experience perspective. This gap has contributed to a limited understanding of the specific needs of postpartum women, which are frequently underestimated or disregarded in the design process.

**Objective:**

This study aims to identify the key factors influencing postpartum women’s use of postpartum exercise rehabilitation mobile applications and to develop corresponding evaluation scales and behavioral models related to their intention to use. The ultimate goal is to promote the health and well-being of postpartum women while offering a theoretical foundation for future research and design practices in relevant domains.

**Methods:**

A mixed-methods approach was employed, integrating qualitative and quantitative research techniques. Initially, user interviews, open-ended questionnaires, and a review of existing literature were conducted to identify the factors that postpartum women consider most important when evaluating postpartum exercise rehabilitation mobile applications. These factors were then synthesized to develop an evaluation scale measuring users’ intention to use. Subsequently, exploratory factor analysis and linear regression analysis were applied to identify the key influencing factors, examine their relationships with intention to use, and construct a behavioral model reflecting the determinants of user intention.

**Results:**

300 valid questionnaires were collected and used for factor analysis and linear regression analysis. The study identified five key factors. The standardized path coefficients between these factors and the intention to use were 0.254, 0.205, 0.198, 0.015, and 0.142, respectively. All factors, except factor 4 (*p* = 0.77), demonstrated statistically significant relationships with intention to use (*p* < 0.05). These findings indicate that exercise safety assurance, physical activity tracking, emotional social support, and health benefits significantly influence users’ intention to use postpartum exercise rehabilitation mobile applications. In contrast, dialogue support does not have a direct effect.

**Conclusion:**

Postpartum women using exercise rehabilitation mobile applications prioritized the practical features of these tools, particularly the safety and efficacy of the exercise regimens and the emotional and social support provided. These findings highlight the importance of aligning functional benefits with users’ health objectives.

## 1 Introduction

The postpartum period encompasses the puerperal phase, typically lasting 4–6 weeks following childbirth, and the subsequent recovery period extends from 6 months to 1 year. During this time, a woman’s physiological functions and hormone levels gradually return to a non-pregnant state. This phase represents a critical period of transformation in a woman’s life, marked by significant emotional vulnerability and profound changes across physiological, psychological, and social dimensions, creating a strong need for comprehensive recovery (Brites-Lagos et al., 2023). Throughout this period, women face a variety of postnatal conditions, including postpartum weight retention, pelvic floor dysfunction, rectal displacement, musculoskeletal pain, and postpartum depression (Tseng et al., 2015; Gebregziabher et al., 2020; de Castro et al., 2022), all of which can substantially affect their quality of life (Norhayati et al., 2015). As a result, postpartum rehabilitation has become a key area of maternal care and research (Skoura et al., 2024), aimed at preserving postpartum women’s physical and mental well-being. Physical exercise, as a cost-effective and straightforward approach to postpartum rehabilitation, has been shown to alleviate several postpartum conditions, significantly improve physical fitness (Tinius et al., 2022), and promote the development of healthy lifestyles (Lee et al., 2021). This makes it an essential component in safeguarding the health and well-being of women during the postpartum period (Bane, 2015). Recent research in physiology, psychology, and public health has demonstrated that exercise supports postpartum recovery by improving cardiovascular function (Mottola, 2002), strengthening pelvic floor muscles (Artymuk and Khapacheva, 2022), and promoting musculoskeletal health (Vladutiu et al., 2015). It also helps alleviate psychological and endocrine conditions such as postpartum depression, insomnia, stress, and role adjustment difficulties (Yang and Chen, 2018). Governments and organizations worldwide have increasingly recognized exercise as an essential recovery tool for women in the postpartum phase (Mottola et al., 2018; Birsner and Gyamfi-Bannerman, 2020; Campos et al., 2021; Brown et al., 2022). In conclusion, postpartum exercise rehabilitation not only serves as an effective means of improving physical health and preventing or treating various dysfunctions, but it also contributes to mental well-being by alleviating anxiety and depression, thereby helping women regain self-confidence and restore both their physical and psychological health.

In recent years, digital health has emerged as an innovative approach that leverages digital information and communication technologies to enhance health outcomes, improve healthcare services, and promote disease prevention. It has made significant contributions to public health development and the self-management of individual health, playing a crucial role in improving population health, enhancing healthcare efficiency and precision, and fostering the personalization of services (Hu et al., 2017; Brewer et al., 2020). mHealth, a key component of digital health, delivers reliable health information and interventions through low-cost, easily accessible methods, effectively promoting public health, particularly in underserved areas such as rural regions (Griffin et al., 2020). This is especially pertinent given the widespread adoption of smartphones and the large-scale deployment of 5G technology. mHealth offers a promising solution for managing, assessing, and supporting postpartum exercise rehabilitation. Research has demonstrated that mobile applications have become a primary tool for pregnant women to access health information and make health decisions (Brown et al., 2020). Among these, fitness and exercise apps are the most frequently used and downloaded health-related mobile applications, with evidence showing their positive influence on physical activity behaviors (Radzi et al., 2020). As a specialized subset, postpartum exercise rehabilitation mobile applications are designed to address the unique physiological and psychological needs of women during the postnatal period. These mobile applications provide flexible, low-cost exercise interventions that are not limited by time or environment, thereby supporting postpartum women in re-establishing a connection with their bodies (McGannon and McMahon, 2022), alleviating psychological stress (Lesser et al., 2023), and achieving various physical health benefits (DiPietro et al., 2019). Given their accessibility and targeted functionality, such mobile applications hold considerable potential for advancing women’s self-empowerment (Wolinsky, 1980), safeguarding postpartum mental health and well-being (Tinius et al., 2022), and improving health outcomes among women in low-income and resource-constrained settings (Tinius et al., 2021; Turner et al., 2023).

However, existing research on mobile applications for postpartum exercise rehabilitation presents several limitations. First, current mobile applications in the physical activity category and related studies often fail to address the specific characteristics of the postpartum population. In particular, there is a lack of mobile applications explicitly designed for postpartum exercise rehabilitation (Yu et al., 2022), which results in the needs of postpartum women being frequently underestimated or overlooked, despite the significant physical and psychological challenges they face during this period (Edie et al., 2021). Second, while existing research on postpartum exercise rehabilitation mobile applications tends to focus on the alleviation of various postpartum conditions (Radzi et al., 2020), the enhancement of women’s motivation to exercise (Tinius et al., 2022), and the functionality of the mobile applications (Tinius et al., 2021), it largely neglects the examination of postpartum women’s intention to use these mobile applications from the perspective of user experience. The intention to use, which refers to a user’s willingness or likelihood to engage with a technology or system in the future, is a crucial predictor of actual usage and reflects the user’s needs and interests in postpartum exercise rehabilitation (Davis, 1989). Additionally, although postpartum rehabilitation mobile applications can offer comprehensive health information and inclusivity, a lack of user-centered design may render these mobile applications ineffective in meeting the needs of the target population (Torous et al., 2018). While some studies have addressed user experience in the context of postpartum exercise rehabilitation, most of these investigations rely on established theories, scales, or literature-based identification of relevant factors. For instance, Rachel et al. employed the Mobile application Rating Scale (MARS) to evaluate postpartum women’s satisfaction with the BumptUp^®^ mobile application (Tinius et al., 2022), and applied the Health Belief Model to examine the barriers to and determinants of maternal physical activity in rural areas (Tinius et al., 2021). Although existing theoretical models have been extensively validated in the broader fields of health behavior and technology adoption, they have not been systematically applied to the specific context of postpartum exercise rehabilitation. Measurement scales derived from these models often lack sensitivity to the postnatal period’s unique physiological and psychological characteristics, rendering them only partially effective in assessing the intention to use mobile applications for postpartum exercise. As a result, such scales tend to be limited in scope and may introduce limitations into the evaluation process. Table 1 provides an overview of detailed studies on user experience in this context.

**Table 1:** Research on user experience related to postpartum exercise rehabilitation mobile application.

Research elements	Sources
Motivational Material, Social Support	France-Ratcliffe et al. (2024)
Technological Affordances, Engagement, Motivational Technology	Kim and Chung (2024)
Barrier Identification and Resolution, Self-Efficacy, Self-Management, Achievable Goal Setting,	Turner et al. (2023)
Social Support	Perera et al. (2023)
Self-Efficacy, Capacity for Customizations, Self-Assessed, Usability, Ease of Navigation	Tinius et al. (2022)
Evidence-Based Exercise Guidance, Personalization, Progress Tracking, Social Support, Symptom Tracking, Usability, Ease of Navigation, Inclusivity	Tucker et al. (2021)
Perceived Severity and Susceptibility, Perceived Barriers, Perceived, Benefit, Cue-to-Action, Self-Efficacy, Social Support, Evidence-Based Exercise Guidance	Tinius et al. (2021)
Effectiveness	Radzi et al. (2020)
Engagement, Risk Perception, Responsibility, Functionality	Willcox et al. (2015)

Therefore, this study draws on the established methodologies of Wang et al. (2022); Wang et al. (2023) and Xing and Jiang (2024), integrating qualitative research approaches, including user interviews, open-ended questionnaires, and literature review, with quantitative techniques such as factor analysis and linear regression. The study will systematically investigate the primary factors influencing postpartum women’s use of postpartum exercise rehabilitation mobile applications and the relationships between these factors and users’ intention to use. Based on these insights, the study aims to develop a targeted evaluation scale and a behavioral model of intention to use. This research holds significant theoretical and practical value by addressing gaps in existing theoretical frameworks, offering a clearer understanding of user behavior, and providing valuable insights to inform the design and optimization of postpartum exercise rehabilitation mobile applications.

## 2 Materials and methods

### 2.1 Research design

**Figure 1:**
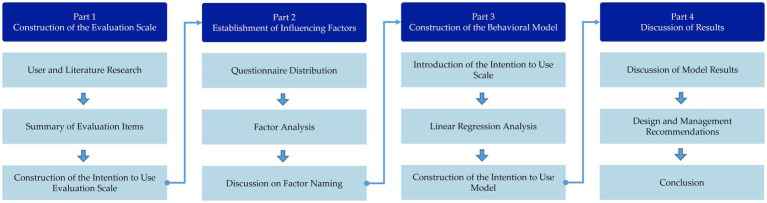
Research process.

Building upon the previous discussion, this study will be conducted in four main parts, with the overall flow of the research depicted in Figure 1.

The first part involves the construction of the evaluation scale. In this stage, the study will begin by collecting postpartum women’s assessments of postpartum exercise rehabilitation mobile applications through a literature review and user interviews. Industry experts will then summarize and review these assessments to finalize the evaluation scale for measuring the intention to use postpartum exercise rehabilitation mobile applications.

The second part focuses on identifying influencing factors. The evaluation scale developed in the previous section will be distributed as a questionnaire in this phase. Reliability analysis, exploratory factor analysis, and confirmatory factor analysis will be employed to identify the key factors influencing the intention to use postpartum exercise rehabilitation mobile applications. Subsequently, the factors will be named based on a thorough literature review concerning the specific items within each factor and the characteristics of postpartum exercise rehabilitation mobile applications, further clarifying the dimensions influencing users’ intention to use.

The third part involves constructing a behavioral model. At this stage, the study will investigate the relationship between the influencing factors identified in the second part and users’ intention to use the mobile application. First, this study will introduce a well-established “Intention to Use” evaluation scale during the questionnaire distribution phase in the second part of the research. It will be distributed alongside the evaluation scale developed in the first part to gather user feedback. Subsequently, exploratory and confirmatory factors will be conducted to ensure that the “Intention to Use” factor has good discriminant validity from the influencing factors established in previous research and that the observed variables within each factor demonstrate strong convergent validity. Following validation, linear regression analysis will be employed to examine the relationship between the influencing factors and intention to use, leading to the construction of a behavioral model of postpartum exercise rehabilitation mobile application usage intention based on the findings.

The fourth part focuses on discussing the results. At this stage, the study will analyze and discuss the relationships between the influencing factors and the intention to use, as identified by the model developed in the third part. Based on this analysis, the study will offer design strategies and management recommendations for designing and optimizing postpartum exercise rehabilitation mobile applications.

### 2.2 Participants

This study was primarily conducted within China, and its representativeness and research value can be justified from several perspectives.

First, China has a substantial population of postpartum women. According to the 2024 World Population Prospects report from the Population Division of the United Nations Department of Economic and Social Affairs, China is projected to have approximately 8.89 million newborns in 2023, ranking second globally (United Nations, 2024). This large cohort provides a robust sample for this study.

Second, China exhibits high Internet penetration and widespread acceptance of health-related mobile applications. By the end of 2024, the number of mobile Internet users had reached 1.17 billion, with a penetration rate exceeding 80%, encompassing nearly all women of childbearing age (Intelligence, 2024). Additionally, surveys indicate that 61% of Chinese citizens have recently used or are currently using mobile health applications, ranking among the highest globally (Statista, 2024). These figures suggest that the adoption and acceptance of postpartum mobile applications among Chinese women are likely equal to or greater than the global average. Therefore, conducting this study in China ensures access to a large and representative sample and enhances the study’s practical relevance and potential generalizability.

Third, the primary approaches to postpartum exercise rehabilitation in China align closely with those recommended by international authorities and widely adopted in developed countries (Health and Excellence, 2006; China, 2017; Syed et al., 2021; Zhu et al., 2022; Zhang et al., 2023). Although certain families in China continue to adhere to traditional cultural beliefs that discourage postpartum women from engaging in physical activity and encourage extended periods of rest (Pillsbury, 1978), the growing dissemination of modern medical knowledge and improvements in living standards have contributed to an increasing number of Chinese women embracing structured exercise to support their recovery (Zhu et al., 2022; Zhang et al., 2023). This trend indicates that the physiological and psychological recovery needs of postpartum women possess a degree of cross-cultural commonality, highlighting the potential universality of evidence-based postpartum rehabilitation practices.

In conclusion, this study highlights that investigating the intention to use postpartum exercise rehabilitation mobile applications in China offers significant advantages regarding universality and sample size. Such research can potentially contribute to optimizing and adopting such mobile applications.

In the user study phase, this research initially conducted in-depth interviews with 71 postpartum women who had participated in postpartum exercise to gather evaluations of the postpartum exercise rehabilitation mobile application. Of the respondents, 44 had used such mobile applications, while 27 had only received postpartum exercise rehabilitation training. This sample size meets the criteria for user interviews (Zhao et al., 2021; Wen et al., 2023). Subsequently, five postgraduate students, who had no vested interest in the study, and two university professors specializing in relevant fields, were invited to review and evaluate the results of the user interviews. Following this initial evaluation, 313 questionnaires were collected through the online platform “Credamo.” After a thorough review to eliminate invalid responses, such as those with unusually short response times, duplicate answers, or missing data, 300 valid questionnaires were retained, yielding a validity rate of 95.85%. This sample size is sufficient for factor analysis (Comrey and Lee, 1992; Price, 1993; Dai et al., 2020; Hair et al., 2021). All respondents were women who had either used the postpartum exercise rehabilitation mobile application or received structured postpartum exercise rehabilitation training. The demographic details of the respondents are presented in Table 2.

**Table 2:** Respondent information from the questionnaire survey.

Title	Option	Frequency	Percentage
Age group	21 ~ 30	93	31.00
	31 ~ 40	158	52.67
	41 ~ 50	29	9.67
	51 ~ 60	20	6.67
Educational background	Junior high school and below	8	2.67
	High school vocational school	15	5.00
	Associate degree	31	10.33
	Bachelor’s degree	168	56.00
	Master’s degree	72	24.00
	Doctoral degree	6	2.00
Occupation	Civil servant	7	2.33
	State-owned enterprise	34	11.33
	Private enterprise	202	67.33
	Public institution	36	12.00
	Foreign-invested enterprise	16	5.33
	Student	5	1.67
Total		300	100.00

### 2.3 Construction of the evaluation scale

**Figure 2:**
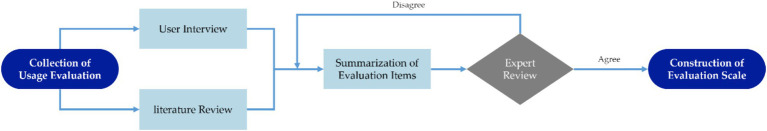
Construction process of the evaluation scale.

The process of constructing the evaluation scale for the intention to use the postpartum exercise rehabilitation mobile application is illustrated in Figure 2. This study draws on the established methodologies of Wang et al. (2022); Wang et al. (2023); Wang et al. (2025), which has been widely applied in various studies focused on user behavior research (Chen et al., 2024; Xing and Jiang, 2024). When the evaluation scales constructed using this method were distributed as questionnaires, the returned data exhibited high reliability and validity, yielding promising results in subsequent data analysis.

During the user research phase, this study utilized structured interviews to gather postpartum women’s evaluations of postpartum exercise rehabilitation mobile applications. The core interview questions were: “What advantages do you believe a postpartum exercise rehabilitation mobile application offers that would make you want to use it?” “What disadvantages do you perceive that would make you reluctant to use such an mobile application?” and “What features or elements should a postpartum exercise rehabilitation mobile application include to encourage your usage?” All participants were fully briefed on the study’s content and purpose before the interview, and their consent was obtained before proceeding. Participants were informed that they could terminate the interview at any time. For participants who had received postpartum exercise rehabilitation training but had not used relevant mobile applications, the study introduced “Kegel Exercise,” a widely used and highly rated mobile application in China, explicitly designed for postpartum women. Participants were familiarized with its functions through guided trial use. Once they had acquired sufficient familiarity with the mobile application, they continued to respond to the interview questions based on their rehabilitation experience and impressions of the mobile application. Ultimately, 427 original evaluations were obtained during the user study phase. To minimize potential bias and skewed data arising from a single source of evaluation, this study also supplemented the user data by collating relevant evaluations of postpartum exercise rehabilitation mobile applications from existing literature. In total, 486 evaluations were collected in the user study phase.

Next, the study consolidated evaluation items derived from the user research phase. Five graduate students with no vested interest in the research were invited to merge evaluation items with similar or identical content to ensure objectivity and reduce potential bias. Following this initial consolidation, two university professors with expertise in postpartum exercise rehabilitation were invited to review the results. In cases where discrepancies arose, the graduate students revised the items until both professors reached complete agreement. The consolidation process yielded 38 items. All negatively worded statements were rephrased into positive formulations to ensure consistency in phrasing. Moreover, to enhance data validity, items with a post-merging frequency of fewer than three responses were excluded from further analysis (Wang et al., 2023). As a result, 28 items were retained for developing the evaluation scale assessing the intention to use postpartum exercise rehabilitation mobile applications. These items are listed in Table 3.

**Table 3:** Summary of evaluation items for user research.

Item	Detailed description	Frequency	Source
Q1	Personalized exercise rehabilitation plan customization	58	Literature Review (Tinius et al., 2021; Turner et al., 2023; Brites-Lagos et al., 2024) and User Survey
Q2	Experts in the relevant field supervise and guide me during exercise	53	Literature Review (Szumilewicz, 2018; Tinius et al., 2021; Brites-Lagos et al., 2023; Lesser et al., 2023; Turner et al., 2023; Jones et al., 2024) and User Survey
Q3	Providing professional and effective exercise methods and techniques	40	Literature Review (Brites-Lagos et al., 2023; Lesser et al., 2023; Brites-Lagos et al., 2024) and User Survey
Q4	Effectively helps me recover from physical injuries caused by pregnancy	32	Literature Review (Tinius et al., 2021; Brites-Lagos et al., 2023; Lesser et al., 2023; Brites-Lagos et al., 2024) and User Survey
Q5	Effectively reduces my weight and helps me achieve a good physique	25	Literature Review (Tinius et al., 2021; Lesser et al., 2023; Tornero-Quiñones et al., 2023; Jones et al., 2024) and User Survey
Q6	Regularly provide feedback on my physical changes	25	Literature Review (Brites-Lagos et al., 2023) and User Survey
Q7	Low-cost or free of charge items	25	User Survey
Q8	Track and record various physical data indicators	23	Literature Review (Tinius et al., 2021) and User Survey
Q9	Flexible exercise duration	23	Literature Review (Tinius et al., 2021; Lesser et al., 2023; Jones et al., 2024) and User Survey
Q10	Equipped with community interaction and communication features	20	Literature Review (Tinius et al., 2021; Lesser et al., 2023; Brites-Lagos et al., 2024; Jones et al., 2024) and User Survey
Q11	Improve physical fitness and restore my level of physical activity	18	Literature Review (Lesser et al., 2023; Brites-Lagos et al., 2024) and User Survey
Q12	Teach me relevant knowledge and precautions regarding postpartum rehabilitation	17	Literature Review (Tinius et al., 2021; Lesser et al., 2023) and User Survey
Q13	Provide dietary and nutrition plans during postpartum rehabilitation	15	User Survey
Q14	Can provide support to motivate me to exercise	14	Literature Review (Radzi et al., 2020; Tinius et al., 2021; Brites-Lagos et al., 2023; Lesser et al., 2023; Jones et al., 2024) and User Survey
Q15	Improve postpartum psychological issues and maintain mental health	13	Literature Review (Tinius et al., 2021; Lesser et al., 2023; Jones et al., 2024) and User Survey
Q16	Equipped with notification and reminder functions to encourage me to exercise	9	Literature Review (Tinius et al., 2021) and User Survey
Q17	Can communicate online with doctors or relevant professionals	9	Literature Review (Tinius et al., 2021; Turner et al., 2023) and User Survey
Q18	Adjust the exercise intensity and plan in real time based on my actual exercise and physical condition	8	Literature Review (Tinius et al., 2021; Brites-Lagos et al., 2023) and User Survey
Q19	Provide corresponding safety measures to reduce my exercise risks	8	Literature Review (Radzi et al., 2020; Tinius et al., 2021; Brites-Lagos et al., 2023; Lesser et al., 2023) and User Survey
Q20	Engage in exercise in a group setting	6	Literature Review (Brites-Lagos et al., 2023; Lesser et al., 2023; Brites-Lagos et al., 2024; Jones et al., 2024) and User Survey
Q21	Help family and friends understand the importance of postpartum exercise	6	Literature Review (Tinius et al., 2021) and User Survey
Q22	Incorporates a reward mechanism	5	User Survey
Q23	Data recording is accurate and effective	5	User Survey
Q24	Able to assess the accuracy of exercise postures and provide corrections	4	User Survey
Q25	Able to protect personal privacy and security	3	User Survey
Q26	Timely update on the latest knowledge and exercise programs related to postpartum rehabilitation	3	User Survey
Q27	Help me develop healthy lifestyle habits	3	Literature Review (Tinius et al., 2021; Lesser et al., 2023) and User Survey
Q28	Can help me gain a sense of pride and confidence	3	Literature Review (Lesser et al., 2023; Jones et al., 2024) and User Survey

To further investigate the intrinsic relationships between various influencing factors and users’ intention to use in subsequent studies, this research selected a well-established “Intention to Use” evaluation scale to gather postpartum women’s feedback on their use of postpartum exercise rehabilitation mobile applications. The chosen scale has demonstrated strong validity in measuring user intention and behavioral attitudes and has been widely cited in numerous studies. The specific content of the questionnaire is presented in Table 4. In adapting this evaluation scale for the study, the wording was modified to fit the context, replacing the research subject with postpartum exercise rehabilitation mobile applications to ensure relevance. The final evaluation scale was created by merging the summarized evaluation scale with the existing “Intention to Use” scale. This final scale will be published online as a seven-point Likert scale to gather user feedback. Before participants could provide input, a participant informed consent form was included at the beginning of the questionnaire. Respondents were required to read the form thoroughly and select the “Agree” option to proceed with the survey. Additionally, a screening question, “Have you used a postpartum exercise rehabilitation mobile application or undergone postpartum exercise rehabilitation training?” was incorporated to ensure that only respondents with relevant experience participated, enhancing the responses’ authenticity and validity.

**Table 4:** Evaluation scale for intention to use.

Latent variable	Observed variable	Literature source
Intention to Use, ITU	If I plan to have a child in the near term, I will use a postpartum exercise rehabilitation mobile application in the near future	Huang and Qian (2021)
	I am willing to use a postpartum exercise rehabilitation mobile application	
	I would recommend that others use a postpartum exercise rehabilitation mobile application	

### 2.4 Data analysis

In this study, an exploratory factor analysis was performed on the data from 300 respondents using SPSS 26.0 software, identifying five key factors. These factors included: exercise safety assurance (comprising items Q1, Q2, Q18, Q19, Q24), physical activity tracking (comprising items Q6, Q8, Q23, Q25, Q26), emotional social support (comprising items Q10, Q20, Q21, Q28), dialogue support (comprising items Q14, Q16, Q22), and health benefits (comprising items Q5, Q11, Q27). The Cronbach’s *α* values for each factor were above 0.7, indicating strong internal consistency and good reliability [68]. Subsequently, a confirmatory factor analysis was conducted using Amos 26.0 software, which confirmed that the factors exhibited satisfactory convergent and discriminant validity. This suggests that the user experience dimensions identified in this study are robust and coherent.

Building on these results, linear regression analysis was performed to examine the relationships between the identified factors and users’ intention to use the postpartum exercise rehabilitation mobile application. The analysis revealed that, except for dialogue support, all other factors directly impacted users’ intention to use the mobile application.

## 3 Results

### 3.1 Factor analysis results

#### 3.1.1 Exploratory factor analysis

In this study, the user evaluation data presented in Table 3 were first subjected to exploratory factor analysis to identify the key elements influencing postpartum women’s intention to use the postpartum exercise rehabilitation mobile application. Upon importing the data into SPSS 26.0 software, six factors with eigenvalues greater than 1 were initially identified, as shown in Table 5. However, the analysis revealed some issues with factor entanglement, where the factor loading coefficients of certain items exceeded 0.4 across multiple factors. This indicated that these items did not exclusively correspond to a single underlying construct but were influenced by various constructs, undermining the distinctiveness between the factors. As a result, the factors failed to meet the criterion of one dimension corresponding to a set of questions, essential for ensuring valid and meaningful factor structures. This suggested that the initial exploratory factor analysis results were invalid. To address this issue, the study adopted a refinement approach based on the methodologies of Joshi et al. (2022), Wei et al. (2020), and Wang et al. (2022); Wang et al. (2023). Specifically, items exhibiting factor entanglement were sequentially deleted, and exploratory factor analyses were conducted after each deletion. This process continued until each observed variable was associated with a single factor, with a factor loading coefficient greater than 0.4. This iterative procedure was crucial for ensuring that the factor structure exhibited both high purity and discriminant validity. Other researchers have widely utilized and validated the approach, demonstrating its scientific rigor and credibility.

**Table 5:** Results of the first exploratory factor analysis.

Item	Factor loading coefficients						Communality
	Factor 1	Factor 2	Factor 3	Factor 4	Factor 5	Factor 6	
Q1	0.693	0.063	0.042	0.123	0.296	−0.001	0.589
Q2	0.683	0.156	0.207	0.096	0.063	0.196	0.585
Q7	0.577	0.077	0.071	0.199	0.056	0.212	0.432
Q9	0.466	0.141	0.514	0.094	−0.064	0.292	0.599
Q18	0.486	0.252	0.107	0.307	0.344	−0.108	0.535
Q19	0.667	0.256	0.031	0.057	0.039	0.204	0.558
Q24	0.567	0.186	0.196	0.225	0.339	−0.048	0.562
Q3	0.120	0.413	0.140	0.569	0.142	0.001	0.548
Q4	0.077	0.471	−0.080	0.477	−0.044	0.332	0.574
Q6	0.054	0.704	0.273	0.247	0.166	−0.038	0.663
Q8	0.245	0.621	−0.016	0.276	0.184	0.145	0.577
Q23	0.369	0.583	0.286	0.025	0.272	0.047	0.634
Q25	0.244	0.553	0.040	0.133	0.219	0.328	0.539
Q26	0.228	0.581	0.334	0.085	0.116	0.153	0.545
Q10	0.240	0.146	0.152	0.165	0.548	0.321	0.532
Q20	0.085	0.256	0.096	0.085	0.657	0.190	0.558
Q21	0.009	0.195	0.413	0.011	0.452	0.477	0.641
Q14	0.061	0.244	0.686	0.128	0.053	0.136	0.571
Q16	0.125	0.023	0.698	0.021	0.276	0.042	0.582
Q22	0.079	0.093	0.805	0.008	0.082	0.014	0.671
Q5	0.052	0.340	0.257	0.680	0.087	0.094	0.664
Q11	0.291	0.288	−0.044	0.657	0.096	0.080	0.617
Q27	0.226	−0.099	0.006	0.762	0.213	0.181	0.720
Q28	0.254	0.094	0.158	0.200	0.688	0.009	0.612
Q12	0.307	0.410	0.373	0.183	0.055	0.275	0.514
Q13	0.200	0.033	0.173	0.351	0.228	0.490	0.486
Q15	0.246	0.185	0.129	0.121	0.139	0.648	0.565
Q17	0.379	0.226	0.017	0.075	0.333	0.318	0.413
Before rotation							
Eigenvalue	9.359	1.974	1.563	1.164	1.049	0.978	
Variance explained (%)	33.426	7.049	5.583	4.157	3.748	3.494	
After rotation							
Eigenvalue	3.462	3.141	2.748	2.677	2.297	1.762	
Variance explained (%)	12.364	11.217	9.813	9.561	8.203	6.294	
KMO and Bartlett’s Test							
KMO	0.931						
Bartlett’s sphericity test	*p* = 0.000						

The results of the exploratory factor analysis were refined after the sequential deletion of items Q3, Q4, Q7, Q9, Q12, Q13, Q15, and Q17. Ultimately, five factors with eigenvalues greater than 1 were identified, with all factor loadings and communalities exceeding 0.4 for each item within the factors. Additionally, Bartlett’s test of sphericity yielded a *p*-value of 0.000 (<0.05), and the Kaiser-Meyer-Olkin (KMO) measure was 0.918 (>0.6), indicating that there were significant differences among the observed variables within each factor and strong correlations between them. These results demonstrate that the exploratory factor analysis was successful and met the requirements for factor validity (Kaiser, 1958). The final results are presented in Table 6. Subsequently, a reliability analysis was conducted, revealing that the Cronbach’s alpha for each factor exceeded 0.7, signifying good internal consistency and reliability of the data used in this study (Eisinga et al., 2013). This further supported the adequacy of the data for subsequent confirmatory factor analysis.

**Table 6:** Results of exploratory factor analysis.

Item	Factor Loading Coefficients					Communality
	Factor 1	Factor 2	Factor 3	Factor 4	Factor 5	
Q1	0.744	0.085	0.18	0.036	0.114	0.608
Q2	0.712	0.165	0.155	0.169	0.066	0.591
Q18	0.537	0.196	0.262	0.085	0.291	0.487
Q19	0.712	0.254	0.01	0.055	0.096	0.584
Q24	0.617	0.172	0.256	0.168	0.213	0.549
Q6	0.053	0.717	0.138	0.241	0.227	0.645
Q8	0.203	0.679	0.193	−0.052	0.298	0.631
Q23	0.38	0.596	0.229	0.263	−0.005	0.621
Q25	0.271	0.623	0.251	0.047	0.106	0.538
Q26	0.224	0.642	0.124	0.311	0.081	0.581
Q10	0.257	0.173	0.624	0.119	0.212	0.544
Q20	0.15	0.265	0.703	0.049	0.051	0.592
Q21	0.042	0.223	0.652	0.378	0.009	0.619
Q28	0.297	0.07	0.64	0.123	0.23	0.57
Q14	0.083	0.268	0.076	0.722	0.13	0.623
Q16	0.13	0.047	0.269	0.703	0.046	0.587
Q22	0.128	0.103	0.075	0.816	−0.023	0.699
Q5	0.088	0.34	0.101	0.27	0.667	0.651
Q11	0.22	0.359	0.078	−0.058	0.712	0.694
Q27	0.214	−0.014	0.203	0.006	0.806	0.737
Before rotation						
Eigenvalue	6.984	1.77	1.316	1.096	0.987	
Variance explained (%)	34.919	8.851	6.58	5.481	4.934	
After rotation						
Eigenvalue	2.869	2.783	2.235	2.228	2.038	
Variance explained (%)	14.345	13.913	11.176	11.142	10.188	
Cronbach α	0.793	0.811	0.727	0.718	0.734	
KMO and Bartlett’s test						
KMO	0.918					
Bartlett’s sphericity test	*p* = 0.000					

#### 3.1.2 Confirmatory factor analysis

A confirmatory factor analysis was conducted to further assess the internal consistency of the observed variables within each factor and the discriminant validity among factors. The results in Table 7 indicate that the standardized factor loadings for all observed variables exceeded 0.5, the average variance extracted (AVE) values for all factors were above 0.36, and the composite reliability (CR) values were greater than 0.6. These findings suggest a strong correspondence between each factor and its associated items and satisfactory convergent validity across the scale (Shevlin and Miles, 1998; Muilenburg and Berge, 2005). In addition, the square root of the AVE for each factor exceeded the correlation coefficients between that factor and all other factors, as shown in Table 8. This demonstrates that each factor is empirically distinct, thereby confirming the discriminant validity of the measurement model. These results indicate that the intention-to-use evaluation scale developed in this study meets established criteria for reliability, convergent validity, and discriminant validity, supporting its applicability for assessing users’ intention to use postpartum exercise rehabilitation mobile applications.

**Table 7:** Results of confirmatory factor analysis.

Factor	Item	Coef.	Std. Error	z	*p*	Std. Estimate	AVE	CR
Factor 1	Q1	1	–	–	–	0.647	0.434	0.793
	Q2	1.158	0.122	9.47	0	0.668		
	Q18	1.011	0.109	9.308	0	0.653		
	Q19	1.028	0.115	8.919	0	0.619		
	Q24	1.229	0.125	9.866	0	0.705		
Factor 2	Q6	1	–	–	–	0.68	0.464	0.812
	Q8	0.885	0.088	10.035	0	0.664		
	Q23	1.009	0.093	10.888	0	0.731		
	Q25	0.926	0.094	9.854	0	0.65		
	Q26	0.904	0.088	10.229	0	0.678		
Factor 3	Q10	1	–	–	–	0.663	0.402	0.728
	Q20	0.935	0.107	8.708	0	0.613		
	Q21	1.004	0.113	8.851	0	0.625		
	Q28	1.077	0.12	8.944	0	0.634		
Factor 4	Q14	1	–	–	–	0.689	0.461	0.719
	Q16	1.006	0.117	8.612	0	0.659		
	Q22	1.152	0.131	8.794	0	0.687		
Factor 5	Q5	1	–	–	–	0.669	0.48	0.734
	Q11	1.197	0.124	9.632	0	0.748		
	Q27	1.021	0.114	8.958	0	0.657		

**Table 8:** Results of discriminant validity analysis.

	Factor 1	Factor 2	Factor 3	Factor 4	Factor 5
Factor 1	*0.659*				
Factor 2	0.585	*0.681*			
Factor 3	0.557	0.572	*0.634*		
Factor 4	0.345	0.446	0.446	*0.679*	
Factor 5	0.492	0.531	0.437	0.231	*0.693*

The italicized diagonal values represent the square roots of the AVE.

#### 3.1.3 Factor naming

The findings of the above analysis confirm that the five factors identified through factor analysis meet established research criteria. Accordingly, this study defines and names each factor based on the content of its associated items, supported by a comprehensive review of relevant literature, to clarify the conceptual dimensions they represent.

Factor 1 comprises five items: Q1, Q2, Q18, Q19, and Q24. These items emphasize the importance of incorporating safety measures into postpartum exercise rehabilitation mobile applications, including developing and adjusting personalized exercise programs. Such measures are intended to prevent physical injuries and avoid worsening postpartum health conditions during use. This is similar to the “Injury Prevention and Control” concept proposed by Ranney et al. and the “Efficacy and Safety” suggested by Dieter et al. Ranney et al. (2022) highlighted the pivotal role of remote monitoring and safety alerts in safeguarding users during exercise. Dieter et al. (2024) emphasized that expert guidance and in-app monitoring can significantly reduce the risk of exercise-related injuries. In addition, the American College of Sports Medicine (ACSM) has consistently emphasized the importance of exercise safety in successive editions of its guidelines for exercise testing and prescription (Medicine, 2013). Based on synthesizing these findings, this study designates Factor 1 as “Exercise Safety Assurance.” This construct refers to applying comprehensive methods to safeguard postpartum women during rehabilitation training and minimize the risk of injury or symptom exacerbation.

Factor 2 comprises five items: Q6, Q8, Q23, Q25, and Q26. These items collectively reflect the mobile application’s capabilities in data collection, feedback provision, accuracy of recorded information, privacy protection, and continuous knowledge updating. These elements address postpartum women’s need for comprehensive data management and individualized feedback regarding their physical condition. This factor aligns with the established “Physical Activity Tracking” concept within the broader framework of physical activity self-regulation (Ehlers and Huberty, 2014). Physical activity tracking involves using technological tools, such as wearable devices and mobile applications, to monitor, record, analyze, and provide feedback on an individual’s exercise behavior (Li et al., 2021). Prior studies have demonstrated that this process supports users in self-assessing their progress (Ehlers and Huberty, 2014) and enhances their health-related knowledge acquisition (Krebs and Duncan, 2015). Based on this conceptual alignment, Factor 2 is designated “Physical Activity Tracking.” It refers to a systematic approach to monitoring and evaluating postpartum physical activity through digital technologies, enabling users to track progress, receive timely feedback, and access personalized health information while ensuring data privacy and integrity.

Factor 3 comprises four items: Q10, Q20, Q21, and Q28. These items focus on fostering a supportive and non-judgmental environment for postpartum women by encouraging interpersonal communication and social support. These items aim to enhance users’ confidence and motivation to engage in physical activity. This aligns with the “Emotional Social Support” concept defined by House, who categorizes social support into four types: emotional, informational, instrumental, and appraisal support. Emotional social support specifically involves expressions of empathy, care, attentive listening, and encouragement, which help individuals feel understood and accepted (House, 1983). Beyond verbal reassurance, it also encompasses the sense of connection and belonging fostered through group interactions and shared experiences (Weiss, 1975; Barrera, 1986). Based on this conceptual correspondence, Factor 3 is labeled “Emotional Social Support” in this study.

Factor 4 comprises three items: Q14, Q16, and Q22. These items reflect mechanisms designed to encourage and motivate postpartum women to engage in physical activity, including regular encouragement, push notifications, and reward-based incentives. These elements closely correspond to the “Dialogue Support” concept outlined by Oinas et al. within the Persuasive Systems Design (PSD) framework, which identifies dialogue support as one of four core system functions. This concept refers to the interactive communication between a system and its user, aimed at promoting and sustaining desired behaviors through responsive feedback (Oinas-Kukkonen and Harjumaa, 2018). Such support simulates human interaction by offering timely reminders, suggestions, praise, and rewards, reinforcing the user’s motivation and commitment to the behavior (Orji and Moffatt, 2018; Oyebode et al., 2021). In light of this alignment, Factor 4 is designated as “Dialogue Support” in this study.

Factor 5 comprises three items: Q5, Q11, and Q27. These items reflect users’ perceptions of the postpartum exercise rehabilitation mobile application as effective in promoting weight loss, restoring physical fitness, and fostering healthy lifestyle habits. These features align with the broader concept of “Health Benefits,” which refers to the positive psychological and physiological outcomes of regular physical activity or structured exercise programs. Such outcomes include reduced risk of chronic illness, improved emotional well-being, and adoption of long-term healthy behaviors (Warburton et al., 2006). Accordingly, Factor 5 was designated health benefits in this study, capturing postpartum women’s expectations and subjective experiences regarding the physical and psychological improvements gained through the mobile application.

### 3.2 Model construction results

#### 3.2.1 Hypothesis formulation

This study intends to employ linear regression analysis to examine further the relationships between the identified influencing factors and the intention to use the postpartum exercise rehabilitation mobile application. A behavioral model will also be constructed to understand better the users’ intention to use the mobile application. Before conducting the data analysis, the study proposes the following hypotheses, derived from the influencing factors identified in Section 3.1:

*H1:* “Exercise Safety Assurance” positively influences postpartum women’s intention to use the postpartum exercise rehabilitation mobile application.

*H2:* “Physical Activity Tracking” positively influences postpartum women’s intention to use the postpartum exercise rehabilitation mobile application.

*H3:* “Emotional Social Support” positively influences postpartum women’s intention to use the postpartum exercise rehabilitation mobile application.

*H4:* “Dialogue Support” positively influences postpartum women’s intention to use the postpartum exercise rehabilitation mobile application.

*H5:* “Health Benefits” positively influences postpartum women’s intention to use the postpartum exercise rehabilitation mobile application.

**Figure 3:**
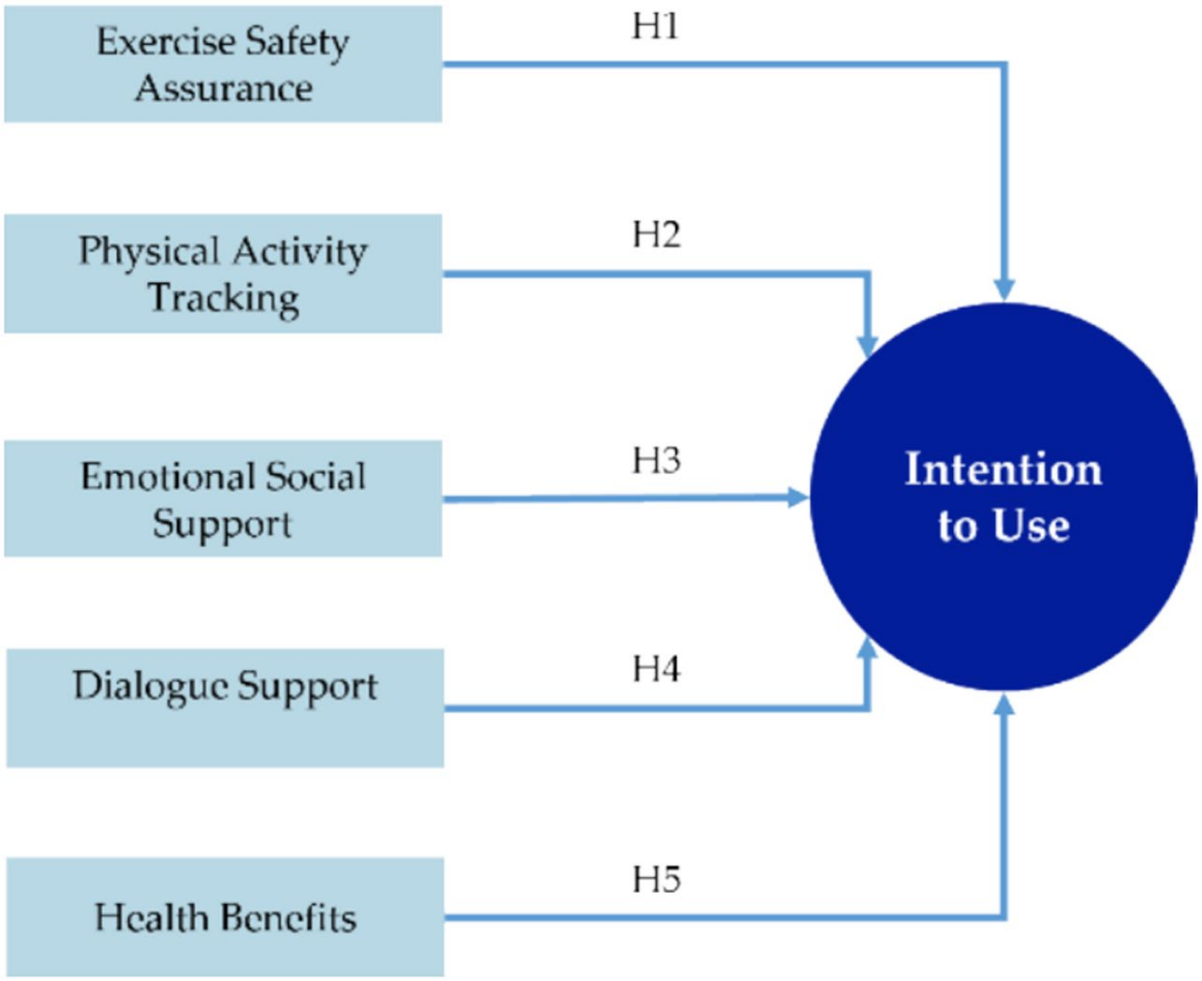
Hypothetical model of users’ intention to use.

The hypothesized model is presented in Figure 3.

For analysis, this study incorporated the well-established dimension of “Intention to Use” alongside the five identified factors to assess postpartum women’s intention to use the mobile application. To ensure that the inclusion of the “Intention to Use” dimension does not affect the other dimensions of the established evaluation scales, the study will combine the five factors with “Intention to Use” and perform both exploratory factor analysis and confirmatory factor analysis before conducting the formal linear regression analysis. This approach aims to verify strong discriminant validity between the six factors and that the items within each factor are appropriately aggregated.

#### 3.2.2 Exploratory factor analysis of the behavioral model

In this study, exploratory factor analysis was employed to assess whether the inclusion of the “Intention to Use” dimension influenced the results of the evaluation scale developed. The findings in Table 9 indicate that the six factors are distinguishable, with the clustering of observed variables within each factor remaining unaffected by the other factors. Additionally, the evaluation scale’s results were not altered. Furthermore, the reliability coefficients for each factor exceeded 0.7, demonstrating satisfactory internal consistency. These findings support the suitability of confirming factor analysis for further validation.

**Table 9:** Exploratory factor analysis results of the behavioral model.

Item	Factor loading coefficients						Communality
	Factor 1	Factor 2	Factor 3	Factor 4	Factor 5	ITU	
Q1	0.745	0.086	0.185	0.036	0.109	0.077	0.616
Q2	0.682	0.13	0.136	0.169	0.056	0.224	0.582
Q18	0.541	0.201	0.276	0.08	0.281	0.102	0.505
Q19	0.702	0.239	0.003	0.063	0.082	0.143	0.582
Q24	0.596	0.136	0.222	0.163	0.202	0.252	0.554
Q6	0.076	0.742	0.159	0.25	0.22	−0.007	0.692
Q8	0.209	0.693	0.205	−0.046	0.29	0.068	0.657
Q23	0.363	0.574	0.219	0.26	−0.021	0.208	0.621
Q25	0.224	0.567	0.206	0.044	0.08	0.369	0.559
Q26	0.203	0.608	0.101	0.312	0.064	0.244	0.583
Q10	0.225	0.143	0.598	0.117	0.2	0.234	0.537
Q20	0.145	0.247	0.668	0.051	0.042	0.169	0.561
Q21	0	0.172	0.606	0.378	−0.009	0.293	0.626
Q28	0.316	0.107	0.685	0.121	0.226	−0.05	0.649
Q14	0.06	0.233	0.058	0.719	0.123	0.19	0.63
Q16	0.155	0.073	0.299	0.705	0.044	−0.08	0.624
Q22	0.126	0.089	0.059	0.812	−0.02	0.072	0.692
Q5	0.067	0.316	0.074	0.272	0.656	0.204	0.656
Q11	0.22	0.362	0.081	−0.059	0.701	0.117	0.694
Q27	0.196	−0.025	0.193	0.005	0.799	0.149	0.737
ITU1	0.227	0.123	0.122	0.095	0.123	0.746	0.662
ITU2	0.235	0.149	0.125	−0.015	0.236	0.752	0.714
ITU3	0.167	0.19	0.327	0.226	0.101	0.5	0.482
Before rotation							
Eigenvalue	7.905	1.826	1.1	1.324	0.98	1.08	
Variance explained (%)	34.368	7.937	4.784	5.756	4.262	4.695	
After rotation							
Eigenvalue	2.876	2.688	2.269	2.283	2.036	2.062	
Variance explained (%)	12.502	11.687	9.865	9.925	8.854	8.966	
Cronbach α	0.793	0.811	0.718	0.727	0.734	0.700	
KMO and Bartlett’s test							
KMO	0.923						
Bartlett’s sphericity test	*p* = 0.000						

#### 3.2.3 Confirmatory factor analysis of the behavioral model

This study conducted confirmatory factor analysis to assess the degree of convergence among the observed variables within each factor and to evaluate the discriminant validity between factors. The results in Tables 10, 11 indicate that the standardized loadings of the observed variables for each factor exceed 0.5, the AVE values are greater than 0.36, and the CR values exceed 0.6. Additionally, the square roots of the AVE for each factor are higher than the correlation coefficients between that factor and the others. These findings suggest that each factor demonstrates strong discriminant validity and that the observed variables within each factor are well-converged. As such, the study is deemed suitable for further linear regression analysis.

**Table 10:** Confirmatory factor analysis results of the behavioral model.

Factor	Item	Coef.	Std. Error	z	*p*	Std. Estimate	AVE	CR
Factor 1	Q1	1	–	–	–	0.641	0.434	0.793
	Q2	1.171	0.124	9.47	0	0.669		
	Q18	1.021	0.11	9.302	0	0.653		
	Q19	1.033	0.116	8.879	0	0.616		
	Q24	1.25	0.126	9.909	0	0.71		
Factor 2	Q6	1	–	–	–	0.669	0.464	0.812
	Q8	0.891	0.091	9.849	0	0.657		
	Q23	1.029	0.095	10.782	0	0.733		
	Q25	0.956	0.097	9.877	0	0.66		
	Q26	0.924	0.091	10.164	0	0.682		
Factor 3	Q10	1	–	–	–	0.666	0.402	0.729
	Q20	0.931	0.106	8.798	0	0.612		
	Q21	1.015	0.112	9.055	0	0.635		
	Q28	1.052	0.118	8.906	0	0.622		
Factor 4	Q14	1	–	–	–	0.689	0.461	0.719
	Q16	1.006	0.117	8.616	0	0.659		
	Q22	1.152	0.131	8.796	0	0.687		
Factor 5	Q5	1	–	–	–	0.671	0.48	0.734
	Q11	1.187	0.122	9.708	0	0.744		
	Q27	1.022	0.113	9.044	0	0.659		
ITU	ITU1	1	–	–	–	0.663	0.447	0.707
	ITU2	1.006	0.105	9.571	0	0.714		
	ITU3	0.95	0.109	8.721	0	0.625		

**Table 11:** Discriminant validity analysis results of the behavioral model.

	Factor 1	Factor 2	Factor 3	Factor 4	Factor 5	ITU
Factor 1	*0.659*					
Factor 2	0.585	*0.681*				
Factor 3	0.557	0.572	*0.634*			
Factor 4	0.345	0.446	0.446	*0.679*		
Factor 5	0.492	0.531	0.437	0.231	*0.693*	
ITU	0.559	0.549	0.525	0.315	0.465	*0.669*

The italicized diagonal values represent the square roots of the AVE.

#### 3.2.4 Linear regression analysis

**Figure 4:**
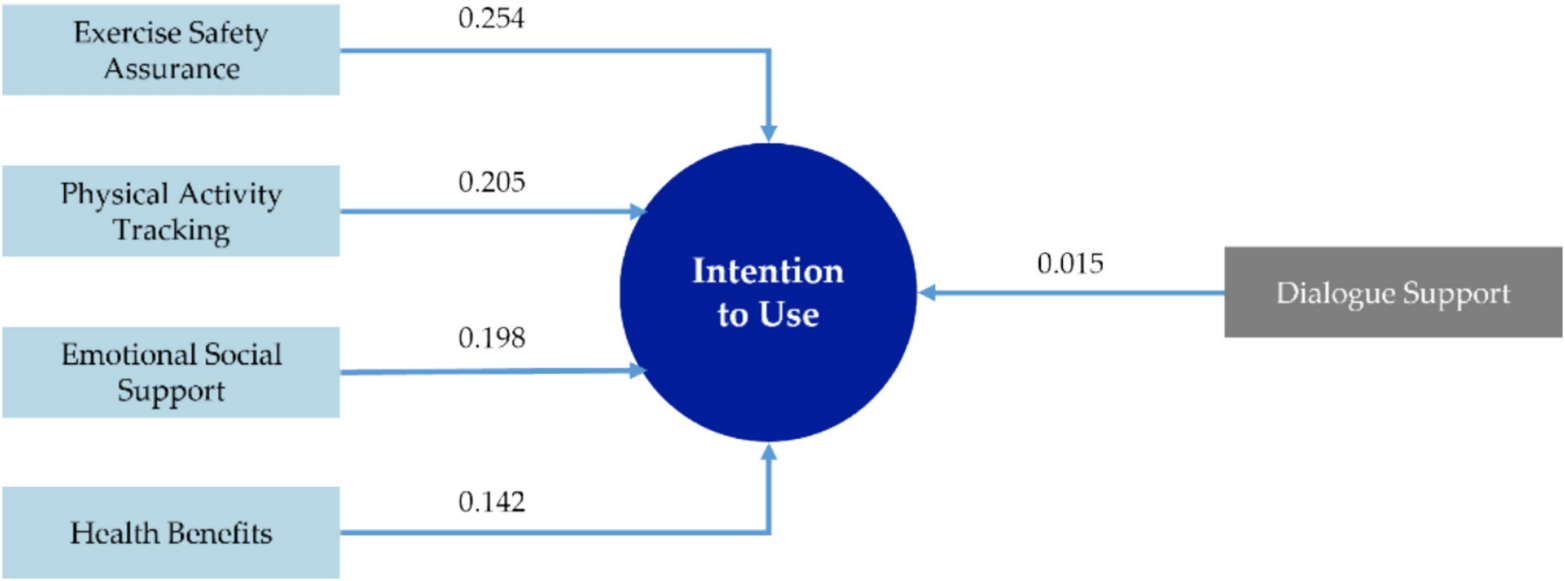
Behavioral model of users’ intention to use the postpartum exercise rehabilitation mobile application.

Building upon the theoretical assumptions outlined in Section 3.2.1, this study further examines the relationship between each factor and the intention to use the postpartum exercise rehabilitation mobile application through linear regression analysis. Factors 1 to 5 were treated as independent variables, with “Intention to Use” as the dependent variable. The results are presented in Table 12. The overall Durbin-Watson (DW) value is 1.906, and the Variance Inflation Factor (VIF) values for each path fall within the range of 0 to 10, indicating that the analysis is valid. The standardized regression coefficients for hypotheses H1 to H5 are 0.254, 0.205, 0.198, 0.015, and 0.142, respectively. The *p*-values for all hypotheses are below 0.05, except for H4, which has a *p*-value of 0.77, indicating that H1, H2, H3, and H5 are supported, while H4 is not. Based on these findings and the factor naming and linear regression analysis results, a behavioral model of users’ intention to use the postpartum exercise rehabilitation mobile application was developed, as shown in Figure 4.

**Table 12:** Results of the linear regression analysis.

Hypothesis	Unstandardized coefficients		Standardized coefficients	t	*p*	Collinearity diagnosis		Result
	B	Standard error	Beta			VIF	Tolerance	
H1	0.260	0.061	0.254	4.288	0.000**	1.800	0.556	Supported
H2	0.208	0.064	0.205	3.256	0.001**	2.033	0.492	Supported
H3	0.196	0.058	0.198	3.35	0.001**	1.802	0.555	Supported
H4	0.011	0.036	0.015	0.293	0.77	1.347	0.743	Rejected
H5	0.135	0.052	0.142	2.613	0.009**	1.523	0.657	Supported

Note: *p < 0.05, ** *p* < 0.01.

## 4 Discussion

### 4.1 Discussion of model results

Based on the results of model construction, this study concluded that four factors, exercise safety assurance, physical activity tracking, emotional social support, and health benefits, significantly influence the intention to use the postpartum exercise rehabilitation mobile application. In contrast, dialogue support did not have a significant effect.

The path coefficient between exercise safety assurance and intention to use was the highest, at 0.254, indicating that exercise safety assurance is a core factor influencing postpartum women’s use of the mobile application. During the postpartum period, women are still undergoing physiological recovery. Fluctuations in hormone levels and reductions in muscle strength increase their vulnerability to injury, heightening their perception of exercise-related risks and potential complications (Soan et al., 2014; Tinius et al., 2021; Inge et al., 2022; Kettle et al., 2022). Mobile applications that provide evidence-based exercise guidance and professional supervision can reduce these perceived risks and significantly enhance exercise self-efficacy, increasing the likelihood of physical activity (Rosenstock, 1974; Bandura, 2004). Research by Evenson et al. also indicates that the perceived safety of exercise instructions substantially improves participation rates among postpartum women (Evenson et al., 2009). Therefore, exercise safety assurance addresses postpartum women’s most pressing safety concerns and functions as a foundational condition for the effectiveness of other features. It is critical in enabling safe participation, supporting habit formation, and strengthening the intention to use the mobile application. However, caution should be exercised to avoid overemphasizing exercise risks and safety warnings within the mobile application. Excessive emphasis on safety may intimidate users, ultimately diminishing their motivation to engage (Witte, 1992). Additionally, as individuals’ health status and birth experiences can vary widely, a one-size-fits-all solution often fails to meet the diverse needs of users (Pescatello, 2014). Exercise programs should be tailored to individual health status, functional capacity, and stage of recovery to minimize unnecessary risks of injury.

Physical activity tracking can directly influence users’ intention to use the postpartum exercise rehabilitation mobile application. This aligns with self-regulation theory (Carver and Scheier, 1982) and the PSD model (Oinas-Kukkonen and Harjumaa, 2018), both of which emphasize that self-monitoring (tracking steps, calories burned) enhances intrinsic motivation and persistence. Exercise tracking and progress management are key design elements for improving behavioral monitoring and feedback, strengthening users’ sense of purpose and commitment to their goals (Carver and Scheier, 1982; Locke and Latham, 2002; Gagnon et al., 2018). These features have been identified as some of the most favored by female exercisers (Ehlers and Huberty, 2014; Li et al., 2021). In the context of postpartum exercise rehabilitation, physical activity tracking can enhance the perceived utility and value of the mobile application. It significantly contributes to the perceived usefulness of the mobile application among postpartum women, thus boosting their motivation to exercise and increasing their intention to use the mobile application. However, concerns regarding data privacy may hinder some users’ willingness to engage with the mobile application (Malhotra et al., 2004; Hernandez et al., 2011). Therefore, it is crucial to implement robust privacy protections during data tracking to alleviate such concerns.

Emotional social support can directly influence users’ intention to use the postpartum exercise rehabilitation mobile application. The companionable and interactive features of the mobile application, coupled with the encouragement and support from family and friends, can make postpartum women feel understood and motivated to engage in physical activity. Social behavior theory suggests that support for family, friends, or peers effectively enhances adherence to health behaviors (Cohen and Wills, 1985). Emotional support is particularly crucial during the postpartum period, when mothers often experience both physical and psychological stress. Empirical studies have shown a positive association between social support and physical activity levels in postpartum women (Bennetter et al., 2023), with cooperation and companionship from family members and peers significantly increasing exercise participation (Evenson et al., 2009). Thus, incorporating social features into mobile applications can enhance user engagement and increase postpartum mothers’ intention to use the mobile application. However, the effectiveness of emotional social support is limited by the user’s social network’s activity level and cultural dynamics. For users who are more isolated or uncomfortable with online sharing, the impact of this support may be reduced (Thoits, 2011). Furthermore, while virtual social interaction can be beneficial, it cannot fully replace the value of real-life companionship, and social features should not be relied upon as a sole means of addressing emotional needs (Rains and Young, 2009). Therefore, it is essential to focus on evidence-based education regarding exercise rehabilitation, which can improve family and friends’ understanding of and support for postpartum women’s behaviors.

The health benefits factor has the smallest path coefficient of 0.135, indicating a weaker effect on the intention to use than the other factors. This may be attributed to health benefits being categorized as an “Outcome Expectation,” representing a more future-oriented cognitive evaluation (Bandura, 2004). As a result, users tend to assign less importance to such long-term outcomes when making decisions (Frederick et al., 2002). Empirical research indicates that the motivational influence of distant outcomes is generally weaker than that of immediate self-efficacy-related experiences. As such, postpartum women, who are uncertain about their future recovery, are more likely to prioritize immediate, visible risk signals or benefits over potential future health improvements. Nonetheless, health benefits remain a crucial factor influencing the adoption of new technologies or products. This aligns with the concept of “Perceived Usefulness” in both the Health Belief Model (Champion and Skinner, 2008) and the Technology Acceptance Model (TAM) (Davis, 1989), which suggests that individuals are more inclined to use a mobile application when they believe it will yield significant benefits. From a postnatal health perspective, exercise has been shown to alleviate symptoms of postnatal depression and anxiety, aid in returning to pre-pregnancy weight, and enhance physical strength (DiPietro et al., 2019; Brites-Lagos et al., 2023). However, caution is necessary to avoid overstating or exaggerating the potential effects of exercise rehabilitation within mobile applications. Overpromising results may lead to unrealistic expectations among postpartum women (Oliver, 1980; Bhattacherjee, 2001), resulting in user disengagement if the promised benefits are not realized.

The linear regression analysis revealed that H4 does not hold, indicating that dialogue support does not directly affect users’ intention to use the mobile application. Several factors may explain this outcome. First, postpartum women often face significant time constraints and prefer quick and convenient access to information (Evenson et al., 2009). As such, frequent interactions with dialogue support may be perceived as an additional burden, conflicting with the user’s demand for simplicity and ease of use (Ng et al., 2024). Second, overly frequent or undifferentiated push notifications can lead to alert fatigue, where users become desensitized to the notifications or choose to block them, thus diminishing their effectiveness in influencing behavior (Mehrotra et al., 2016). Furthermore, encouragement, reminders, and rewards are external incentives. Existing research suggests that users’ behavior and intention to use are primarily driven by intrinsic motivation (personal interest, values, and satisfaction) and the tool’s inherent usefulness and usability, rather than by additional interactive features (Cerasoli et al., 2014; Ng et al., 2024). The impact of external incentives is considerably reduced if users lack interest in the activity or do not perceive it as contributing to their goals. Self-Determination Theory (SDT) also posits that excessive reliance on external incentives can undermine intrinsic motivation (Deci et al., 1999). Consequently, balancing external incentives with inherent motivation is crucial when designing postpartum exercise rehabilitation mobile applications. For example, incorporating progressive rewards can enhance users’ self-efficacy and sense of achievement, while personalized reminder frequencies can help avoid repetitiveness and prevent feelings of coercion.

Additionally, as the sample in this study predominantly consists of participants from China, the results may be influenced to some extent by traditional Chinese cultural values, particularly in the areas of “Exercise Safety Assurance” and “Emotional Social Support.” In the traditional Chinese “postnatal confinement” culture, new mothers are often encouraged to rest at home and avoid strenuous physical activity to promote recovery and prevent postnatal illnesses. Family and friends usually discourage any intention to engage in physical exercise (Zhang et al., 2023). As a result, Chinese postpartum women may be susceptible to concerns regarding exercise safety and emotional support from family members. This may have led to a heightened influence of these factors on their intention to use the mobile application in this study. However, existing research suggests that the “postnatal confinement” culture is not unique to China. Many other cultures, including those in Southeast Asia (Withers et al., 2018), Latin America (Chapman and Coups, 1999), and West Africa (Dennis et al., 2007), have similar postnatal resting and isolation practices, albeit under different names. While the specifics of these traditions vary, they share a common concern for the physical vulnerability of postpartum women. It can be inferred that the “Exercise Safety Assurance” factor is also crucial in these cultural contexts. Providing evidence-based guidance and monitoring safe exercise remains central to promoting user adoption across cultures. Similarly, while the sources of emotional support for postpartum women, such as family members, spouses, friends, and professional services, may vary across cultural contexts (McLeish et al., 2021; Qi et al., 2022; Stumbar and Minor, 2023), the emphasis of “Emotional Social Support” on familial and community encouragement reflects a universal human need for social connection. Prior research has consistently shown that robust emotional support enhances psychological well-being and promotes behavioral adherence among postpartum women, regardless of cultural background (Khademi and Kaveh, 2024). The sources of such support can be adapted and expanded to fit different sociocultural settings. Consequently, the findings of this study are considered to have a degree of cross-cultural applicability.

Overall, postpartum women using exercise rehabilitation mobile applications were primarily concerned with the practical attributes of the mobile application, particularly the safety and efficacy of the exercises and the emotional and social support it provided. The findings emphasize that aligning the mobile application’s practical benefits and users’ personal health goals is a key driver of usage behavior. This alignment significantly boosted users’ self-efficacy and enhanced their overall perception of the mobile application’s usefulness, strengthening their intention to use it. In contrast, dialogue support did not significantly influence the intention to use, indicating that postpartum users preferred low-interaction information access rather than two-way communication that demands additional time and effort. Simplicity, directness, and efficiency should remain the main design principles for postpartum women. Furthermore, although the results of this study are generalizable, differences in behavioral norms and sources of social support across cultures should be acknowledged. Future research could employ the same scales and methods to compare multiple cultural contexts, thereby validating these factors’ cross-cultural stability and relative influence. This approach would enhance the artistic sensitivity and generalizability of the model.

### 4.2 Design and management recommendations

#### 4.2.1 Recommendations based on exercise safety assurance

An examination of the specific items included in the exercise safety assurance indicates that postpartum women prefer the involvement of a professional in customizing and monitoring their exercise program. Consequently, postpartum exercise rehabilitation mobile applications should integrate experts or experienced practitioners in fields such as exercise science and postpartum rehabilitation. These professionals should form a multidisciplinary coaching and health assessment team to provide high-quality, evidence-based exercise guidance for postpartum women.

First, the expert team should personalize the exercise program for postpartum women based on their body characteristics and postnatal injury status. To facilitate postpartum exercise rehabilitation, mobile applications should allow first-time users to input relevant personal information, including delivery details, desired recovery outcomes, and postnatal injury status. Second, to prevent sports injuries resulting from improper movements, the mobile application should provide instructional videos, demonstrated and explained by professionals, when users encounter new exercises or training routines. The mobile application should also incorporate cameras and sensors to monitor users’ exercise postures in real time and offer corrective feedback, thereby enhancing user trust and improving the overall experience. Finally, postpartum exercise rehabilitation mobile applications should incorporate regular assessments of users’ exercise performance and recovery status, enabling timely adjustments to the intensity, frequency, and type of exercise. Thereby enhancing rehabilitation outcomes and reducing exercise-related risks.

#### 4.2.2 Recommendations based on physical activity tracking

An in-depth analysis of the components within physical activity tracking reveals that accurate data collection and feedback, timely knowledge updates, and data security are key factors in enhancing postpartum women’s intention to use the program. First, the mobile application should track key indicators such as exercise duration, calorie expenditure, workout intensity, and weight changes, presenting these results to users through intuitive visualization charts at different stages. This will enable users to perceive the effectiveness of the mobile application in improving postnatal symptoms and enhancing physical fitness, thereby boosting their self-efficacy and increasing their motivation to continue using the mobile application.

Second, the mobile application should promptly integrate and deliver the latest rehabilitation advice and exercise tips, ensuring all content aligns with authoritative medical standards and current research findings. This will enhance the mobile application’s credibility and encourage users to rely on it for long-term support. Additionally, given the significant amount of personal and exercise-related data stored on the platform, it is essential to implement robust data encryption and identity verification mechanisms to protect users’ privacy and safeguard data security.

#### 4.2.3 Recommendations based on emotional social support

In this study, emotional social support primarily refers to mutual encouragement among postpartum women and their recognition and support from friends and family. Analysis of this dimension suggests that establishing an online communication platform and facilitating group exercise activities are effective strategies for enhancing interaction among postpartum users. Therefore, postpartum exercise rehabilitation mobile applications should include an online community feature that fosters a safe, inclusive, and supportive environment. This can help prevent psychological harm caused by weight-related stigma or stereotypical judgments of postpartum body image. Within this community, users can share experiences related to postpartum life and rehabilitation and initiate or join group activities. Such peer interaction has the potential to boost self-confidence and further motivate sustained participation in physical activity.

Moreover, the mobile application should address the lack of understanding and discouragement from friends and relatives regarding postpartum exercise, which may arise due to local cultural practices. To this end, the mobile application could provide educational videos and readings about postpartum exercise rehabilitation, help family members and friends correct misconceptions, and offer them tools to provide positive and sustained support for postpartum women’s exercise behaviors.

#### 4.2.4 Recommendations based on health benefits

In this study, health benefits emphasize the positive impact of postpartum exercise rehabilitation mobile applications on reducing postpartum weight, improving physical fitness, and encouraging the adoption of a healthy lifestyle. To achieve these objectives, mobile applications must offer well-structured, medically-supported exercise programs that encompass a variety of exercise forms and progressively tailored training goals. By guidelines provided by the World Health Organization (WHO) and the American College of Obstetricians and Gynecologists (ACOG), postpartum women are advised to avoid sedentary behavior and, when physically able, engage in at least 150 min of moderate-intensity aerobic exercise per week (Brites-Lagos et al., 2023). This should be complemented by moderate muscle-strengthening and stretching exercises. As recovery progresses, exercise frequency, intensity, and duration should gradually increase.

The mobile application should also offer guidance on healthy routines and meal management to help users develop a sustainable and healthy lifestyle throughout their recovery.

For detailed design and managerial recommendations, see Table 13.

**Table 13:** Design and management recommendations.

Factor	Recommendations
Exercise safety assurance	1. Establish a multidisciplinary team of postnatal exercise rehabilitation experts. 2. Tailor exercise programs to individual needs and conditions. 3. Provide professional explanations and demonstrations during the initial training session. 4. Monitor exercise sessions in real-time, offering prompt corrections for incorrect movements. 5. Adjust the exercise program regularly based on ongoing physical recovery assessments.
Physical activity tracking	1. Implement real-time tracking and recording of physiological and exercise-related data. 2. Present the data using intuitive visual representations. 3. Ensure timely updates of rehabilitation advice and exercise methods based on the latest medical guidelines. 4. Strengthen data encryption and identity verification processes.
Emotional social support	1. Establish an online community. 2. Provide accessible videos and educational materials on postnatal rehabilitation exercises.
Health benefits	1. Offer a comprehensive, medically-supported exercise rehabilitation program. 2. Design a tailored rest and nutrition plan.

## 5 Conclusion

### 5.1 Research conclusion

With the growing attention to the needs of postpartum women and the rapid advancement of mobile health technology, this study addresses the gap in research on the user experience of postpartum rehabilitation mobile applications. It explores the key factors influencing postpartum women’s use of exercise rehabilitation mobile applications from a user experience perspective, employing both qualitative and quantitative research methods. The study developed an evaluation scale and a behavioral model to assess users’ intention to use the postpartum exercise rehabilitation mobile application, offering corresponding design and managerial recommendations. The primary factors influencing postpartum women’s use of these mobile applications were exercise safety assurance, physical activity tracking, emotional and social support, and health benefits. Dialogue support was found to have no direct effect on users’ intention to use the mobile application.

This suggests that postpartum women prioritize the practical attributes of such mobile applications, highlighting the significance of self-efficacy and outcome expectations in behavioral psychology. Additionally, this finding underscores the importance of aligning functional benefits with user goals in designing postpartum mobile applications. This study contributes to the theoretical foundation of mobile health products in postpartum rehabilitation and is a reference for cross-disciplinary research integrating psychology, public health, and interaction design.

### 5.2 Limitations and prospects

Although this study is pioneering in developing an evaluation scale and behavioral model to assess the intention to use a postpartum exercise rehabilitation mobile application and identify the direct factors influencing users’ intention to use it, several limitations remain.

First, since the research for this study was primarily conducted in China, the findings may be influenced by traditional Chinese cultural norms surrounding the puerperium. While the results and recommendations share some cultural commonalities, differences in postpartum behavior across cultures should be considered. Therefore, future studies could compare the same scales and methods across urban and rural contexts, varying income levels, and diverse cultures to assess the factors’ cross-cultural stability and relative impact, thus enhancing the cultural sensitivity and generalizability of the model.

Secondly, in developing the user experience evaluation scale, this study predominantly relied on self-reported data, such as user interviews, which may introduce subjectivity into the findings. Although efforts were made to supplement this with a comprehensive literature review, potential biases still exist. Future studies could incorporate more objective data on user behavior, such as mobile application usage logs or professional monitoring tools like eye-tracking devices. This would help corroborate the self-reported data and improve the validity and reliability of the evaluation scale.

Finally, while the model developed in this study highlighted the direct positive effects of exercise safety assurance, physical activity tracking, emotional social support, and health benefits on postpartum women’s intention to use the mobile application, the study’s cross-sectional nature limits the model’s comprehensiveness. Several relevant factors, such as affordability, time constraints, lack of time, and fatigue, previously identified in the literature as influencing postpartum physical activity, were not included. Therefore, future research could enhance the model of intention to use postpartum exercise rehabilitation mobile applications by expanding the sample size, broadening the measurement dimensions, and adopting a longitudinal design. Such efforts would contribute to a more robust theoretical framework and offer stronger practical guidance for designing and implementing mobile health interventions in postpartum rehabilitation.

## Data Availability

The datasets for this article are not publicly available due to concerns regarding participant anonymity. Requests to access the datasets should be directed to dr530@jiangnan.edu.cn.

## References

[ref1] Artymuk N. V. Khapacheva S. Y. (2022). Device-assisted pelvic floor muscle postpartum exercise programme for the management of pelvic floor dysfunction after delivery. J. Matern. Fetal Neonatal Med. 35, 481–485. doi: 10.1080/14767058.2020.1723541, PMID: 32019378

[ref2] Bandura A. (2004). Health promotion by social cognitive means. Health Educ. Behav. 31, 143–164. doi: 10.1177/1090198104263660, PMID: 15090118

[ref3] Bane S. M. (2015). Postpartum exercise and lactation. Clin. Obstet. Gynecol. 58, 885–892. doi: 10.1097/GRF.0000000000000143, PMID: 26398298

[ref4] Barrera M. Jr. (1986). Distinctions between social support concepts, measures, and models. Am. J. Community Psychol. 14, 413–445. doi: 10.1007/BF00922627

[ref5] Bennetter K. E. Richardsen K. R. Vøllestad N. K. Jenum A. K. Robinson H. S. Mdala I.. (2023). Associations between social support and physical activity in postpartum: a Norwegian multi-ethnic cohort study. BMC Public Health 23:702. doi: 10.1186/s12889-023-15507-z, PMID: 37069637 PMC10111809

[ref6] Bhattacherjee A. (2001). Understanding information systems continuance: an expectation-confirmation model. MIS Q. 25, 351–370. doi: 10.2307/3250921

[ref7] Birsner M. L. Gyamfi-Bannerman C. (2020). Physical activity and exercise during pregnancy and the postpartum period ACOG committee opinion summary, number 804. Obstet. Gynecol. 135, E178–E188. doi: 10.1097/AOG.0000000000003772 32217980

[ref8] Brewer L. C. Fortuna K. L. Jones C. Walker R. Hayes S. N. Patten C. A.. (2020). Back to the future: achieving health equity through health informatics and digital health. JMIR Mhealth Uhealth 8:e14512. doi: 10.2196/14512, PMID: 31934874 PMC6996775

[ref9] Brites-Lagos C. Maranhao C. Szumilewicz A. Santos-Rocha R. (2024). Development and validation of the physical exercise program "active mums" for postpartum recovery: mobile application of the CReDECI-2 guidelines. BMC Pregnancy Childbirth 24:378. doi: 10.1186/s12884-024-06387-1, PMID: 38769520 PMC11103992

[ref10] Brites-Lagos C. Ramos L. Szumilewicz A. Santos-Rocha R. (2023). Feasibility of a supervised postpartum exercise program and effects on maternal health and fitness parameters-pilot study. Healthcare 11:2801. doi: 10.3390/healthcare11202801, PMID: 37893875 PMC10606677

[ref11] Brown H. M. Bucher T. Collins C. E. Rollo M. E. (2020). A review of pregnancy apps freely available in the Google play store. Health Promot. J. Austr. 31, 340–342. doi: 10.1002/hpja.270, PMID: 31225924

[ref12] Brown W. J. Hayman M. Haakstad L. A. Lamerton T. Mena G. P. Green A.. (2022). Australian guidelines for physical activity in pregnancy and postpartum. J. Sci. Med. Sport 25, 511–519. doi: 10.1016/j.jsams.2022.03.008, PMID: 35418334

[ref13] Campos M. D. S. B. Buglia S. Colombo C. S. S. D. S. Buchler R. D. D. Brito A. S. X. D. Mizzaci C. C.. (2021). Posicionamento sobre Exercícios Físicos na Gestação e no Pós-Parto–2021. Arq. Bras. Cardiol. 117, 160–180. doi: 10.36660/abc.20210408, PMID: 34320089 PMC8294738

[ref14] Carver C. S. Scheier M. F. (1982). Control theory: A useful conceptual framework for personality–social, clinical, and health psychology. Psychol. Bull. 92, 111–135. doi: 10.1037/0033-2909.92.1.111, PMID: 7134324

[ref15] Cerasoli C. P. Nicklin J. M. Ford M. T. (2014). Intrinsic motivation and extrinsic incentives jointly predict performance: a 40-year meta-analysis. Psychol. Bull. 140, 980–1008. doi: 10.1037/a0035661, PMID: 24491020

[ref16] Champion V. L. Skinner C. S. (2008). The health belief model. In Glanz K. Rimer B. K. Viswanath K. (Eds.), Health behavior and health education: Theory, research, and practice 4th edn, 45–65. San Francisco, CA: Jossey-Bass/Wiley.

[ref17] Chapman G. B. Coups E. J. (1999). Time preferences and preventive health behavior: acceptance of the influenza vaccine. Med. Decis. Mak. 19, 307–314. doi: 10.1177/0272989X9901900309, PMID: 10424837

[ref18] Chen J. Pan L. Zhou R. Jiang Q. (2024). Shaping and optimizing the image of virtual city spokespersons based on factor analysis and entropy weight methodology: A cross-sectional study from China. Systems 12:44. doi: 10.3390/systems12020044

[ref19] China. (2017). *Specifications for National Basic Public Health Services (Third Edition)*. Available online at: http://www.nhc.gov.cn/jws/s3578/201703/d20c37e23e1f4c7db7b8e25f34473e1b.shtml [Accessed February 28th, 2017].

[ref20] Cohen S. Wills T. A. (1985). Stress, social support, and the buffering hypothesis. Psychol. Bull. 98, 310–357. doi: 10.1037/0033-2909.98.2.310 3901065

[ref21] Comrey A. L. Lee H. B. (1992). A first course in factor analysis. New York: Psychology Press.

[ref22] Dai H. M. Teo T. Rappa N. A. Huang F. J. C. (2020). Explaining Chinese university students’ continuance learning intention in the MOOC setting: A modified expectation confirmation model perspective. Comput. Educ. 150:103850. doi: 10.1016/j.compedu.2020.103850, PMID: 40353240

[ref23] Davis F. D. (1989). Perceived usefulness, perceived ease of use, and user acceptance of information technology. MIS Q. 13, 319–340. doi: 10.2307/249008

[ref24] de Castro R. Antunes R. Mendes D. Szumilewicz A. Santos-Rocha R. (2022). Can group exercise programs improve health outcomes in pregnant women? An updated systematic review. Int. J. Environ. Res. Public Health 19:4875. doi: 10.3390/ijerph19084875, PMID: 35457743 PMC9024782

[ref25] Deci E. L. Koestner R. Ryan R. M. (1999). A meta-analytic review of experiments examining the effects of extrinsic rewards on intrinsic motivation. Psychol. Bull. 125, 627–668. doi: 10.1037/0033-2909.125.6.627, PMID: 10589297

[ref26] Dennis C.-L. Fung K. Grigoriadis S. Robinson G. E. Romans S. Ross L. (2007). Traditional postpartum practices and rituals: a qualitative systematic review. Womens Health 3, 487–502. doi: 10.2217/17455057.3.4.487, PMID: 19804024

[ref27] Dieter V. Janssen P. Krauss I. (2024). Efficacy of the mHealth-based exercise intervention re. Flex for patients with knee osteoarthritis: pilot randomized controlled trial. JMIR Mhealth Uhealth 12:e54356. doi: 10.2196/54356, PMID: 39250181 PMC11420596

[ref28] DiPietro L. Evenson K. R. Bloodgood B. Sprow K. Troiano R. P. Piercy K. L.. (2019). Benefits of physical activity during pregnancy and postpartum: an umbrella review. Med. Sci. Sports Exerc. 51, 1292–1302. doi: 10.1249/MSS.0000000000001941, PMID: 31095086 PMC6527310

[ref29] Edie R. Lacewell A. Streisel C. Wheeler L. George E. Wrigley J.. (2021). Barriers to exercise in postpartum women: A mixed-methods systematic review. J. Women's Pelvic Health Phys. Ther. 45, 83–92. doi: 10.1097/JWH.0000000000000201

[ref30] Ehlers D. K. Huberty J. L. (2014). Middle-aged women’s preferred theory-based features in mobile physical activity mobile applications. J. Phys. Act. Health 11, 1379–1385. doi: 10.1123/jpah.2012-0435, PMID: 24368818

[ref31] Eisinga R. Te Grotenhuis M. Pelzer B. (2013). The reliability of a two-item scale: Pearson, Cronbach, or spearman-Brown? Int. J. Public Health 58, 637–642. doi: 10.1007/s00038-012-0416-3 23089674

[ref32] Evenson K. R. Aytur S. A. Borodulin K. (2009). Physical activity beliefs, barriers, and enablers among postpartum women. J. Women's Health 18, 1925–1934. doi: 10.1089/jwh.2008.1309, PMID: 20044854 PMC2828187

[ref33] France-Ratcliffe M. Christie H. E. Blunden S. Opie R. S. Chua E. Karimi N.. (2024). Evaluating a multi-behavioural home-based intervention for reducing depressive symptoms in postnatal women: the food, move, sleep (FOMOS) for postnatal mental health randomised controlled trial protocol. Contemp. Clin. Trials 136:107383. doi: 10.1016/j.cct.2023.107383, PMID: 37935305

[ref34] Frederick S. Loewenstein G. O’donoghue T. (2002). Time discounting and time preference: A critical review. J. Econ. Lit. 40, 351–401. doi: 10.1257/jel.40.2.351

[ref35] Gagnon J.-C. Fortier M. McFadden T. Plante Y. (2018). Investigating the behaviour change techniques and motivational interviewing techniques in physical activity counselling sessions. Psychol. Sport Exerc. 36, 90–99. doi: 10.1016/j.psychsport.2018.02.002

[ref36] Gebregziabher N. K. Netsereab T. B. Fessaha Y. G. Alaza F. A. Ghebrehiwet N. K. Sium A. H. (2020). Prevalence and associated factors of postpartum depression among postpartum mothers in central region, Eritrea: a health facility based survey. BMC Public Health 20, 1–10. doi: 10.1186/s12889-020-09676-4, PMID: 33109137 PMC7590801

[ref37] Griffin J. B. Struempler B. Funderburk K. Parmer S. M. Tran C. Wadsworth D. D. (2020). My quest, a community-based mHealth intervention to increase physical activity and promote weight loss in predominantly rural-dwelling, low-income, Alabama women. Fam. Community Health 43, 131–140. doi: 10.1097/FCH.0000000000000251, PMID: 32079969

[ref38] Hair J. F. Jr. Hult G. T. M. Ringle C. M. Sarstedt M. (2021). A primer on partial least squares structural equation modeling (PLS-SEM). Thousand Oaks, CA: Sage Publications.

[ref39] Health and Excellence. (2006). *Postnatal care up to 8 weeks after birth.* National Institute for Health and Clinical Excellence. 32065741

[ref40] Hernandez B. Montaner T. Sese F. J. Urquizu P. (2011). The role of social motivations in e-learning: how do they affect usage and success of ICT interactive tools? Comput. Hum. Behav. 27, 2224–2232. doi: 10.1016/j.chb.2011.07.001

[ref41] House J. S. (1983). Work stress and social support. Series on occupational stress. Reading, MA: Addison-Wesley.

[ref42] Hu H. Galea S. Rosella L. Henry D. (2017). Big data and population health: focusing on the health impacts of the social, physical, and economic environment. Epidemiology 28, 759–762. doi: 10.1097/EDE.0000000000000711, PMID: 28682850

[ref43] Huang Y. Qian L. (2021). Understanding the potential adoption of autonomous vehicles in China: the perspective of behavioral reasoning theory. Psychol. Mark. 38, 669–690. doi: 10.1002/mar.21465

[ref44] Inge P. Orchard J. J. Purdue R. Orchard J. W. (2022). Exercise after pregnancy. Aust. J. Gen. Pract. 51, 117–121. doi: 10.31128/AJGP-09-21-6181, PMID: 35224572

[ref45] Intelligence (2024). The Mobile economy China 2025. London, UK: GSMA.

[ref46] Jones H. M. Orr J. Whelan M. E. Oyebode O. (2024). An exploration of pregnancy and postpartum content on Instagram: A content analysis of health and exercise focused accounts. Women Birth 37:101632. doi: 10.1016/j.wombi.2024.101632, PMID: 38971136

[ref47] Joshi M. A. Krishnappa P. Prabhu A. V. (2022). Faculty satisfaction and perception regarding emergency remote teaching: An exploratory study. Med J. Armed Forces India 79, S258–S266. doi: 10.1016/j.mjafi.2022.04.005 35702712 PMC9186517

[ref48] Kaiser H. F. (1958). The varimax criterion for analytic rotation in factor analysis. Psychometrika 23, 187–200. doi: 10.1007/BF02289233

[ref49] Kettle V. E. Madigan C. D. Coombe A. Graham H. Thomas J. J. Chalkley A. E.. (2022). Effectiveness of physical activity interventions delivered or prompted by health professionals in primary care settings: systematic review and meta-analysis of randomised controlled trials. BMJ 376:e068465. doi: 10.1136/bmj-2021-068465, PMID: 35197242 PMC8864760

[ref50] Khademi K. Kaveh M. H. (2024). Social support as a coping resource for psychosocial conditions in postpartum period: a systematic review and logic framework. BMC Psychol. 12:301. doi: 10.1186/s40359-024-01814-6, PMID: 38807228 PMC11131291

[ref51] Kim H. S. Chung M. Y. (2024). A motivational technology perspective on the use of smart wrist-worn wearables for postpartum exercise and weight management. Health Commun. 40, 268–282. doi: 10.1080/10410236.2024.2343472, PMID: 38644619

[ref52] Krebs P. Duncan D. T. (2015). Health app use among US mobile phone owners: a national survey. JMIR Mhealth Uhealth 3:e4924. doi: 10.2196/mhealth.4924, PMID: 26537656 PMC4704953

[ref53] Lee R. Thain S. Tan L. K. Teo T. Tan K. H. (2021). Asia-Pacific consensus on physical activity and exercise in pregnancy and the postpartum period. BMJ Open Sport Exerc. Med. 7:e000967. doi: 10.1136/bmjsem-2020-000967, PMID: 34055384 PMC8130752

[ref54] Lesser I. A. Nienhuis C. P. Hatfield G. L. (2023). Moms on the move: A qualitative exploration of a postpartum group exercise program on physical activity behaviour at three distinct time points. Int. J. Qual. Stud. Health Well Being 18:2172793. doi: 10.1080/17482631.2023.2172793, PMID: 36710424 PMC9888496

[ref55] Li C. Chen X. Bi X. (2021). Wearable activity trackers for promoting physical activity: a systematic meta-analytic review. Int. J. Med. Inform. 152:104487. doi: 10.1016/j.ijmedinf.2021.104487, PMID: 34020170

[ref56] Locke E. A. Latham G. P. (2002). Building a practically useful theory of goal setting and task motivation: A 35-year odyssey. Am. Psychol. 57, 705–717. doi: 10.1037/0003-066X.57.9.705, PMID: 12237980

[ref57] Malhotra N. K. Kim S. S. Agarwal J. (2004). Internet users' information privacy concerns (IUIPC): the construct, the scale, and a causal model. Inf. Syst. Res. 15, 336–355. doi: 10.1287/isre.1040.0032, PMID: 19642375

[ref58] McGannon K. R. McMahon J. (2022). (Re) storying embodied running and motherhood: a creative non-fiction approach. Sport Educ. Soc. 27, 960–972. doi: 10.1080/13573322.2021.1942821

[ref59] McLeish J. Harvey M. Redshaw M. Alderdice F. (2021). A qualitative study of first time mothers’ experiences of postnatal social support from health professionals in England. Women Birth 34, e451–e460. doi: 10.1016/j.wombi.2020.10.012, PMID: 33153952 PMC8396053

[ref60] Medicine (2013). ACSM's guidelines for exercise testing and prescription. Philadelphia, PA: Lippincott Williams & Wilkins.

[ref61] Mehrotra A. Pejovic V. Vermeulen J. Hendley R. Musolesi M. (2016). "My phone and me: understanding people's receptivity to mobile notifications", in: *Proceedings of the 2016 CHI conference on human factors in computing systems*), 1021–1032.

[ref62] Mottola M. F. (2002). Exercise in the postpartum period: practical mobile applications. Curr. Sports Med. Rep. 1, 362–368. doi: 10.1249/00149619-200212000-00010, PMID: 12831685

[ref63] Mottola M. F. Davenport M. H. Ruchat S.-M. Davies G. A. Poitras V. J. Gray C. E.. (2018). 2019 Canadian guideline for physical activity throughout pregnancy. Br. J. Sports Med. 52, 1339–1346. doi: 10.1136/bjsports-2018-100056, PMID: 30337460

[ref64] Muilenburg L. Y. Berge Z. L. (2005). Student barriers to online learning: A factor analytic study. Distance Educ. 26, 29–48. doi: 10.1080/01587910500081269

[ref65] Ng W. Y. Lau N. Y. Lee V. V. Vijayakumar S. Leong Q. Y. Ooi S. Q. D.. (2024). Shaping adoption and sustained use across the maternal journey: qualitative study on perceived usability and credibility in digital health tools. JMIR Hum. Factors 11:e59269. doi: 10.2196/59269, PMID: 39352732 PMC11480679

[ref66] Norhayati M. Hazlina N. N. Asrenee A. Emilin W. W. (2015). Magnitude and risk factors for postpartum symptoms: a literature review. J. Affect. Disord. 175, 34–52. doi: 10.1016/j.jad.2014.12.041, PMID: 25590764

[ref67] Oinas-Kukkonen H. Harjumaa M. (2018). “Persuasive systems design: key issues, process model and system features” in Routledge handbook of policy design. eds. Howlett M. Mukherjee I. (New York: Routledge), 87–105.

[ref68] Oliver R. L. (1980). A cognitive model of the antecedents and consequences of satisfaction decisions. J. Mark. Res. 17, 460–469. doi: 10.1177/002224378001700405

[ref69] Orji R. Moffatt K. (2018). Persuasive technology for health and wellness: state-of-the-art and emerging trends. Health Informatics J. 24, 66–91. doi: 10.1177/1460458216650979, PMID: 27245673

[ref70] Oyebode O. Ndulue C. Mulchandani D. A. Zamil Adib A. Alhasani M. Orji R. (2021). "Tailoring persuasive and behaviour change systems based on stages of change and motivation", in: *Proceedings of the 2021 CHI conference on human factors in computing systems*), 1–19.

[ref71] Perera M. Hawk G. S. Nagpal T. S. Tinius R. A. (2023). Social support for exercise from pregnancy to postpartum and the potential impact of a mobile application: A randomized control pilot trial in southern United States. Prev. Med. Rep. 36:102485. doi: 10.1016/j.pmedr.2023.102485, PMID: 37954963 PMC10637991

[ref72] Pescatello L. S. (2014). ACSM's guidelines for exercise testing and prescription. Philadelphia, PA: Lippincott Williams & Wilkins.

[ref73] Pillsbury B. L. (1978). “Doing the month”: confinement and convalescence of Chinese women after childbirth. Soc. Sci. Med. B Med. Anthropol. 12, 11–22. doi: 10.1016/0160-7987(78)90003-0 565536

[ref74] Price B. (1993). A first course in factor analysis by Andrew L. Comrey; Howard B. Lee. Technometrics 35:453. doi: 10.4324/9781315827506, PMID: 39964225

[ref75] Qi W. Liu Y. Lv H. Ge J. Meng Y. Zhao N.. (2022). Effects of family relationship and social support on the mental health of Chinese postpartum women. BMC Pregnancy Childbirth 22:65. doi: 10.1186/s12884-022-04392-w, PMID: 35078423 PMC8787939

[ref76] Radzi C. Jenatabadi H. S. Samsudin N. (2020). mHealth apps assessment among postpartum women with obesity and depression. Healthcare 8:72. doi: 10.3390/healthcare8020072, PMID: 32225114 PMC7349810

[ref77] Rains S. A. Young V. (2009). A meta-analysis of research on formal computer-mediated support groups: examining group characteristics and health outcomes. Hum. Commun. Res. 35, 309–336. doi: 10.1111/j.1468-2958.2009.01353.x

[ref78] Ranney M. L. Stettenbauer E. Delgado M. K. Yao K. A. Orchowski L. M. (2022). Uses of mHealth in injury prevention and control: a critical review. Curr. Epidemiol. Rep. 9, 273–281. doi: 10.1007/s40471-022-00312-w, PMID: 36404873 PMC9644389

[ref79] Rosenstock I. M. (1974). Historical origins of the health belief model. Health Educ. Monogr. 2, 328–335. doi: 10.1177/109019817400200403 299611

[ref80] Shevlin M. Miles J. N. (1998). Effects of sample size, model specification and factor loadings on the GFI in confirmatory factor analysis. Personal. Individ. Differ. 25, 85–90. doi: 10.1016/S0191-8869(98)00055-5

[ref81] Skoura A. Billis E. Papanikolaou D. T. Xergia S. Tsarbou C. Tsekoura M.. (2024). Diastasis recti abdominis rehabilitation in the postpartum period: A scoping review of current clinical practice. Int. Urogynecol. J. 35, 491–520. doi: 10.1007/s00192-024-05727-1, PMID: 38340172 PMC11023973

[ref82] Soan E. J. Street S. J. Brownie S. M. Hills A. P. (2014). Exercise physiologists: essential players in interdisciplinary teams for noncommunicable chronic disease management. J. Multidiscip. Healthc. 7, 65–68. doi: 10.2147/JMDH.S55620, PMID: 24511238 PMC3913503

[ref83] Statista (2024). *Share of health app users in selected countries as of 2024*. Available online at: https://www.statista.com/forecasts/1181436/share-of-health-app-users-by-country (Accessed November 19, 2024).

[ref84] Stumbar S. E. Minor S. (2023). Postpartum care up to one year after pregnancy: A systematic review and Meta-analysis. Am. Fam. Physician 108, 506–508, PMID: 37983703

[ref85] Syed H. Slayman T. Thoma K. D. (2021). ACOG committee opinion no. 804: physical activity and exercise during pregnancy and the postpartum period. Obstet. Gynecol. 137, 375–376. doi: 10.1097/AOG.0000000000004266, PMID: 33481513

[ref86] Szumilewicz A. (2018). Who and how should prescribe and conduct exercise programs for pregnant women? Recommendation based on the European educational standards for pregnancy and postnatal exercise specialists. Dev. Period Med. 22, 107–112. doi: 10.34763/devperiodmed.20182202.107112, PMID: 30056396 PMC8522896

[ref87] Thoits P. A. (2011). Mechanisms linking social ties and support to physical and mental health. J. Health Soc. Behav. 52, 145–161. doi: 10.1177/0022146510395592, PMID: 21673143

[ref88] Tinius R. A. Blankenship M. M. Colao A. M. Hawk G. S. Perera M. Schoenberg N. E. (2022). A pilot study on the impact of the BumptUp® Mobile app on physical activity during and after pregnancy. Sustain. For. 14:12801. doi: 10.3390/su141912801, PMID: 37840967 PMC10574187

[ref89] Tinius R. Duchette C. Beasley S. Blankenship M. Schoenberg N. (2021). Obstetric patients and healthcare providers perspectives to inform Mobile app Design for Physical Activity and Weight Control during Pregnancy and postpartum in a rural setting. Int. J. Womens Health 13, 405–432. doi: 10.2147/ijwh.S296310, PMID: 33953614 PMC8092851

[ref90] Tornero-Quiñones I. Gallego M. R. Molina-López J. Sierra-Robles Á. Sáez-Padilla J. (2023). Physical activity association with body image in postpartum women. Psychol. Soc. Educ. 15, 37–44. doi: 10.21071/pse.v15i2.15817

[ref91] Torous J. Nicholas J. Larsen M. E. Firth J. Christensen H. (2018). Clinical review of user engagement with mental health smartphone apps: evidence, theory and improvements. BMJ Ment. Health 21, 116–119. doi: 10.1136/eb-2018-102891, PMID: 29871870 PMC10270395

[ref92] Tseng P.-C. Puthussery S. Pappas Y. Gau M.-L. (2015). A systematic review of randomised controlled trials on the effectiveness of exercise programs on Lumbo pelvic pain among postnatal women. BMC Pregnancy Childbirth 15, 1–12. doi: 10.1186/s12884-015-0736-4 26612732 PMC4661954

[ref93] Tucker L. Villagomez A. C. Krishnamurti T. (2021). Comprehensively addressing postpartum maternal health: a content and image review of commercially available mobile health apps. BMC Pregnancy Childbirth 21:311. doi: 10.1186/s12884-021-03785-7, PMID: 33879089 PMC8059182

[ref94] Turner J. Clanchy K. Vincze L. (2023). Telehealth interventions for physical activity and exercise participation in postpartum women: A quantitative systematic review. Prev. Med. 167:107413. doi: 10.1016/j.ypmed.2022.107413, PMID: 36603606

[ref95] United Nations. (2024). *World Population Prospects 2024*. Available online at: https://population.un.org/wpp/ (Accessed July 18, 2024).

[ref96] Vladutiu C. J. Evenson K. R. Jukic A. M. Herring A. H. (2015). Correlates of self-reported physical activity at 3 and 12 months postpartum. J. Phys. Act. Health 12, 814–822. doi: 10.1123/jpah.2014-0147, PMID: 25157810 PMC4564345

[ref97] Wang Z. Deng R. Jiang Q.-L. (2023). Exploration and study of the factors influencing users’ adoption of games for fitness behavior. Int. J. Hum.–Comput. Interact. 39, 3461–3472. doi: 10.1080/10447318.2022.2097985

[ref98] Wang Z. Jiang Q. Li Z. (2022). How to promote online education through educational software—an analytical study of factor analysis and structural equation modeling with Chinese users as an example. Systems 10:100. doi: 10.3390/systems10040100

[ref99] Wang Z. Wang Z. Deng R. (2025). Assessment scale and behavioral model construction for AI chat information retrieval and processing service systems based on behavioral reasoning theory—taking ChatGPT-like tools as an example. Int. J. Hum.–Comput. Interact. 41, 3031–3052. doi: 10.1080/10447318.2024.2331855

[ref100] Warburton D. E. Nicol C. W. Bredin S. S. (2006). Health benefits of physical activity: the evidence. CMAJ 174, 801–809. doi: 10.1503/cmaj.051351, PMID: 16534088 PMC1402378

[ref101] Wei W. Cao M. Jiang Q. Ou S.-J. Zou H. (2020). What influences Chinese consumers’ adoption of battery electric vehicles? A preliminary study based on factor analysis. Energies 13:1057. doi: 10.3390/en13051057

[ref102] Weiss R. (1975). Loneliness: The experience of emotional and social isolation. Cambridge, MA: MIT Press.

[ref103] Wen X. Wang Y. Yue X. Zhu F. Zhu M. (2023). "Nftdisk: visual detection of wash trading in nft markets", in: *Proceedings of the 2023 CHI conference on human factors in computing systems*), 1–15.

[ref104] Willcox J. C. van der Pligt P. Ball K. Wilkinson S. A. Lappas M. McCarthy E. A.. (2015). Views of women and health professionals on mHealth lifestyle interventions in pregnancy: A qualitative investigation. JMIR Mhealth Uhealth 3, e99–e67. doi: 10.2196/mhealth.4869, PMID: 26510886 PMC4704935

[ref105] Withers M. Kharazmi N. Lim E. (2018). Traditional beliefs and practices in pregnancy, childbirth and postpartum: A review of the evidence from Asian countries. Midwifery 56, 158–170. doi: 10.1016/j.midw.2017.10.019, PMID: 29132060

[ref106] Witte K. (1992). Putting the fear back into fear appeals: the extended parallel process model. Commun. Monogr. 59, 329–349. doi: 10.1080/03637759209376276

[ref107] Wolinsky F. D. (1980). The sociology of health: Principles, professions, and issues. Boston: Little, Brown.

[ref108] Xing J. Jiang Q. (2024). Factors influencing user experience in AI chat systems–a satisfaction study based on factor analysis and linear regression. Kybernetes. 0368-492X. doi: 10.1108/K-10-2023-2237

[ref109] Yang C.-L. Chen C.-H. (2018). Effectiveness of aerobic gymnastic exercise on stress, fatigue, and sleep quality during postpartum: A pilot randomized controlled trial. Int. J. Nurs. Stud. 77, 1–7. doi: 10.1016/j.ijnurstu.2017.09.009, PMID: 28950158

[ref110] Yu H. He J. Wang X. Yang W. Sun B. Szumilewicz A. (2022). A comparison of functional features of Chinese and US mobile apps for pregnancy and postnatal care: a systematic app store search and content analysis. Front. Public Health 10:826896. doi: 10.3389/fpubh.2022.826896, PMID: 35252100 PMC8891489

[ref111] Zhang X. Zuo X. Matheï C. Aertgeerts B. Afnan M. Li T.. (2023). Impact of a postpartum care rehabilitation program to prevent postpartum depression at a secondary municipal hospital in Qingdao China: a cross-sectional study. BMC Pregnancy Childbirth 23:239. doi: 10.1186/s12884-023-05547-z, PMID: 37041524 PMC10088113

[ref112] Zhao T. Cui J. Hu J. Dai Y. Zhou Y. (2021). Is artificial Intelligence customer service satisfactory? Insights based on microblog data and user interviews. Cyberpsychol. Behav. Soc. Netw. 25, 110–117. doi: 10.1089/cyber.2021.0155, PMID: 34935458

[ref113] Zhu H. Zhang D. Gao L. Liu H. Di Y. Xie B.. (2022). Effect of pelvic floor workout on pelvic floor muscle function recovery of postpartum women: protocol for a randomized controlled trial. Int. J. Environ. Res. Public Health 19:11073. doi: 10.3390/ijerph191711073, PMID: 36078788 PMC9517758

